# Restoring dryland old fields with native shrubs and grasses: Does facilitation and seed source matter?

**DOI:** 10.1371/journal.pone.0205760

**Published:** 2018-10-18

**Authors:** Shauna M. Uselman, Jay Davison, Owen W. Baughman, Benjamin W. Sullivan, W. Wally Miller, Elizabeth A. Leger

**Affiliations:** 1 Department of Natural Resources and Environmental Science, University of Nevada, Reno, Nevada, United States of America; 2 University of Nevada Cooperative Extension, Fallon, Nevada, United States of America; 3 The Nature Conservancy, Burns, Oregon, United States of America; USDA Forest Service Rocky Mountain Research Station, UNITED STATES

## Abstract

Restoration of agricultural fields is challenging, especially in arid and semi-arid ecosystems. We conducted experiments in two fields in the Great Basin, USA, which differed in cultivation history and fertility. We tested the effects of different levels of functional diversity (planting grasses and shrubs together, vs. planting shrubs alone), seed source (cultivars, local or distant wild-collections), and irrigation regime (spring or fall and spring) on restoration outcomes. We sowed either: 1) grasses and shrubs in year one, 2) shrubs only, in year one, 3) grasses in year one with herbicide, shrubs in year two, or 4) shrubs alone in year two, after a year of herbicide. We irrigated for two years and monitored for three years. Shrub emergence was highest in the lower fertility field, where increasing functional diversity by seeding grasses had a neutral or facilitative effect on shrub emergence. In the higher fertility field, increasing functional diversity appeared to have a neutral to competitive effect. After declines in shrub densities after irrigation ceased, these effects did not persist. Grasses initially suppressed or had a neutral effect on weeds relative to an unseeded control, but had neutral or facilitative effects on weeds relative to shrub-only seeding. Initially, commercial grasses were either equivalent to or outperformed wild-collected grasses, but after irrigation ceased, commercial grasses were outperformed by wild-collected grasses in the higher fertility field. Local shrubs initially outperformed distant shrubs, but this effect did not persist. Fall and spring irrigation combined with local shrubs and wild-collected grasses was the most successful strategy in the higher fertility field, while in the lower fertility field, irrigation timing had fewer effects. Superior shrub emergence and higher grass persistence indicated that the use of wild and local seed sources is generally warranted, whereas the effects of functional diversity and irrigation regime were context-dependent. A bet-hedging approach that uses a variety of strategies may maximize the chances of restoration success.

## Introduction

Abandonment of agricultural practices in dryland ecosystems is becoming increasingly common worldwide [1–3]. For example, in Nevada, a state centrally-located in the North American Great Basin, there was a 41% reduction in farmland between 1992 and 2012 [4]. Agricultural land abandonment often occurs due to changing social and economic priorities, and is associated with numerous ecological consequences [1,5,6]. Restoring native vegetation to abandoned farmland, which is often dominated by weeds and agricultural plants, is desirable to ameliorate these effects and enhance a variety of ecosystem services, such as wildlife habitat, reduced erosion, and increased biodiversity. Agricultural legacies include altered plant community composition and cover as well as changes to soil characteristics such as soil organic matter content and nutrient availability [7–9], and these changes make restoring abandoned old fields particularly challenging. However, the existence of irrigation infrastructure in former agriculture fields can allow for the temporary irrigation of newly seeded restoration areas, a treatment which is not available in most wildland seedings. This early addition of water may assist plants to overcome critical barriers to establishment during germination, emergence and early stages of development when plants may be more prone to water stress [10,11].

Another factor that can improve restoration outcomes in arid and semiarid ecosystems (hereafter, drylands) is the use of seeds that are adapted to site conditions [12]. The role of local adaptation in the restoration of old fields is currently of great interest, with most work to date in tallgrass prairie [13–15]. Local, wild-collected grasses, for example, may be more successful in old fields because they are well adapted to the local climatic conditions [16,17]. In altered old fields, however, while climatic conditions may be “local” (i.e., more similar to origins of wild-collected seeds), old field soil conditions are decidedly “non-local” (i.e., altered from natural conditions due to agricultural legacies). Alternatives to wild-collected seeds include commercially-sourced grass cultivars, which are readily available and cost-effective, and have frequently been selected for favorable agronomic traits such as forage yield and quality, high seed production and/or harvestability, high/fast rates of germination, and/or seedling vigor under agronomic conditions [18]. Given these traits, cultivars may be more productive, and thus more competitive in invaded old-fields, relative to locally-sourced grasses. However, it is also possible that cultivars may not be as adapted to harsh soil or climatic conditions at restoration sites (i.e., the “cultivar vigor hypothesis” vs. the “local adaptation hypothesis,” *sensu* [19]). Thus, the relative performance of local seeds vs. commercially-available cultivars in old fields may be different than results observed in other dryland systems [20].

Plant-plant interactions, both competitive and facilitative, can also play a large role in restoration outcomes [21]. In water-limited ecosystems, grasses have been shown to facilitate shrub and tree establishment during restoration [22–24]. At the same time, competition with weeds is a major barrier to native plant establishment during restoration of degraded areas in dryland environments [25]. Seeding more competitive grasses at the onset of restoration can be desirable to help suppress weeds and reduce soil erosion [26], and this strategy can be compatible with shrub establishment [27]. Separating the seeding of grasses and shrubs over time (seeding grasses first, followed by shrubs in subsequent years) also allows for the use of broadleaf specific herbicides during the grass-only planting phases, which can reduce weeds [26], and may enhance the potential for facilitative effects from grasses. However, plant-plant interactions can be highly context-dependent, and increasing functional diversity by planting both grasses and shrubs during old field restoration, rather than planting just shrubs, could also increase the likelihood of competitive interactions [26,28].

The stress-gradient hypothesis predicts that plant facilitation is more likely to occur in more stressful environments [29], and experimental results generally support this hypothesis [30–34]. Although dryland ecosystems are generally considered stressful, water application during old field restoration may alleviate resource limitation and alter the balance between facilitation and competition. For example, our previous research in dryland old-field ecosystems indicated that grasses were competitive with shrubs when seeded and irrigated at relatively high levels [26]. In this earlier work, commercially-sourced grasses were initially very successful when well-watered, and as a result, shrub emergence and early establishment was diminished. While facilitation may occur under conditions with limited soil resources, where benefits from neighboring plants (such as improved water relations or higher nutrient availability) exceed the costs of competition, competition for light may predominate under more productive conditions [35,36]. The shift between facilitation and competition likely depends on many factors, including environmental conditions, species identity and functional type, developmental stage, and local adaptation [33,37–40], and it is important to understand how plant-plant interactions shift from facilitative to neutral or competitive in irrigated old fields.

Here, we seek to improve our understanding of treatments that can increase restoration success in dryland old-field environments, and improve upon the results of our last experiment. First, we ask how functional diversity affects restoration outcomes, specifically asking whether seeded grasses can suppress weeds and facilitate shrubs during restoration. To promote facilitation, we reduced the total water addition and seeding rates from those used in a previous study [26], with the aim of lessening the potential for grass-shrub competition. Second, we investigated the role of seed source in restoration success, and asked whether the balance between facilitation and competition would depend on seed origin [39], and we included multiple seed sources for grasses (commercially available cultivars or wild-collected seeds) and shrubs (locally- or distantly-sourced wild-collected seeds). Finally, we were interested in the role of resource availability on restoration outcomes, and asked whether watering regimes interacted with functional diversity and seed source during old field restoration, and whether these results were consistent in sites with underlying differences in soil fertility. We used existing irrigation infrastructure to test plant responses to water availability by varying the timing and amount of total water addition, using spring only irrigation or fall plus spring irrigation, at two old field sites differing in soil fertility and time since cultivation. We reduced water application, relative to our previous experiment, in an attempt to provide enough water to improve native plant establishment without stimulating a major weed response, as previous observed [26]. We altered water timing as well as overall addition because in North American cold deserts, summer drought extends into the fall season in some years but not others, and rainfall during the fall season is often a cue for different patterns of plant phenology and/or development [41]. We addressed the following specific questions:

1) Does functional diversity help or hinder shrub emergence and establishment?
  1. Does seeding both grasses and shrubs result in lower weed abundance or weed height?
  2. Do results depend on seed origins?

We investigate these questions under two scenarios: (1) shrubs and grasses seeded together in the first year, and (2) shrubs seeded in the second year, one year after grass seeding and weed control, comparing both of these scenarios to shrub-only seedings. We expected greater weed suppression under the grass seeding treatments, and expected these grass seedings to facilitate shrub establishment.

2) Does seed source affect restoration outcomes?

We compared emergence and establishment of commercially-sourced vs. wild-collected perennial grasses, and wild-collected shrubs originating from either more local or distant seed sources, and we also asked how seed source affected weed abundance. Simultaneous manipulation allowed us to ask if there were potential interactions among seed sources of these two functional groups. Because of agricultural legacy effects in these old-fields, we expected commercially-sourced grass cultivars to perform well during irrigation treatments, but for their performance to decline after irrigation ceased. We expected more locally-collected shrubs to outperform more distant collections. Because of the potential for commercially-available grasses to grow large quickly, we expected greater weed suppression from these sources, but for similar reasons, lower shrub survival, especially for non-local shrubs.

3) How does resource availability affect restoration outcomes?

We tested this question by comparing plant abundance under two different irrigation regimes (spring only vs. fall and spring irrigation), and by conducting our experiments in two restoration sites that differ in soil fertility due to differences in agricultural histories. We expected fall and spring irrigation treatments to outperform spring-only irrigation, as many of the native species in this region can germinate in the fall, or require a cold/wet seed stratification for germination. Further, we expected greater restoration success in our lower-fertility field site, due to reduced weed competition, and expected that seed sources would have strong, but possibly opposite, impacts on restoration outcomes in our two sites.

Through these questions, we aimed to identify restoration treatments that result in the highest densities of grass and shrub establishment, and identify a strategy that was effective in old field sites with different land use history. For all questions, we present our results for two restoration stages: (1) plant emergence and early seedling development over the first two years after seeding (2015–2016), and (2) plant survival and establishment during the third year of the study (2017), after irrigation treatments ceased.

## Methods

### Site description

The study was located along the East Walker River approximately 45 km upstream of Yerington, Nevada, USA. Two former agricultural fields in need of ecological restoration were selected within the salt desert zone of the Great Basin Desert. Separated by approximately 1.4 km, the “North Field” (38°40’N, 118°58’W; Elevation 1463 m) and the “South Field” (38°39’N, 118°58’W; Elevation 1469 m) were historically leveled, tilled, and cultivated for the production of *Medicago sativa* L. (alfalfa). These sites differ in their recent history of cultivation, and represent the two main types of old fields requiring restoration in this region: fields transitioning directly from production to restoration (North Field), or abandoned fields needing active restoration to return native shrub communities (South Field). The North Field was under alfalfa production for a decade or more until the summer of 2013, while the South Field had not been in production for at least a decade. Prior to the start of the experiment, the North Field contained abundant residual alfalfa plants, while the South Field contained sparse alfalfa plants, and common agricultural weeds were present at both sites (S1 Appendix.) Both sites were regularly irrigated during cultivation, and the basic irrigation infrastructure remained at the onset of this experiment.

**Fig 1:**
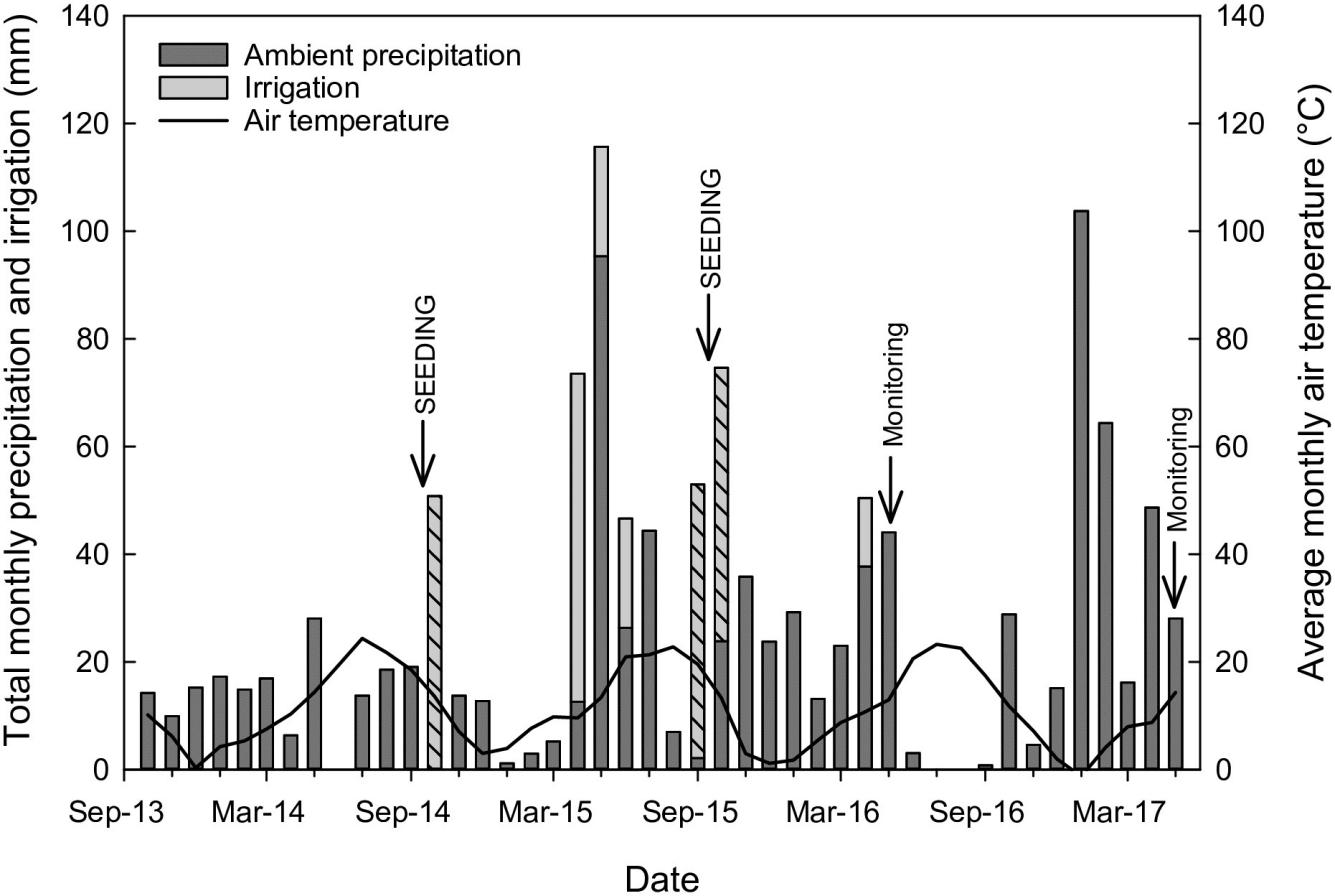
Ambient precipitation (mm), irrigation added (mm), and average monthly air temperature (°C) during the study. Fall irrigation is indicated with hash marks. Seeding and monitoring dates are indicated with arrows.

Historic annual precipitation at the sites averaged 179 mm (7.04 in) for the period of 1980–2010 (PRISM Climate Group, Oregon State University, http://prism.oregonstate.edu, accessed 15 Oct. 2016). Over the experimental period, total precipitation was 174 (97%), 223 (125%), and 234 mm (131% of average) during the 2014, 2015, and 2016 water years (Oct. 1 –Sept. 30), respectively (Fig 1). For the period from Oct. 1, 2016 through the end of May 2017 when we performed our last monitoring, total precipitation was 309 mm, or 223% above the average for that period. Soils at both sites are classified in the Fallon series (coarse-loamy, mixed, superactive, nonacid, mesic Oxyaquic Torrifluvents) (USDA Web Soil Survey (http://websoilsurvey.nrcs.usda.gov; accessed 5 February 2016)). The North Field soil is somewhat finer textured than the South Field soil, and consistent with textural differences and more recent alfalfa production, it is a more fertile site, especially in terms of N availability (S2 Appendix). Native plant communities in nearby surrounding salt desert natural areas are dominated by shrubs, including *Sarcobatus vermiculatus* (Hook.) Torr. (black greasewood), *Atriplex confertifolia* (Torr. & Frém.) S. Watson (shadscale saltbush), *Atriplex canescens* (Pursh) Nutt. (fourwing saltbush), *Atriplex torreyi* (S. Watson) S. Watson (Torrey’s saltbush), *Artemisia tridentata* Nutt. (big sagebrush), *Picrothamnus desertorum* Nutt. (bud sagebrush), and *Ericameria nauseosa* (Pall. ex Pursh) G.L. Nesom & Baird (rubber rabbitbrush).

### Experimental design

**Fig 2:**
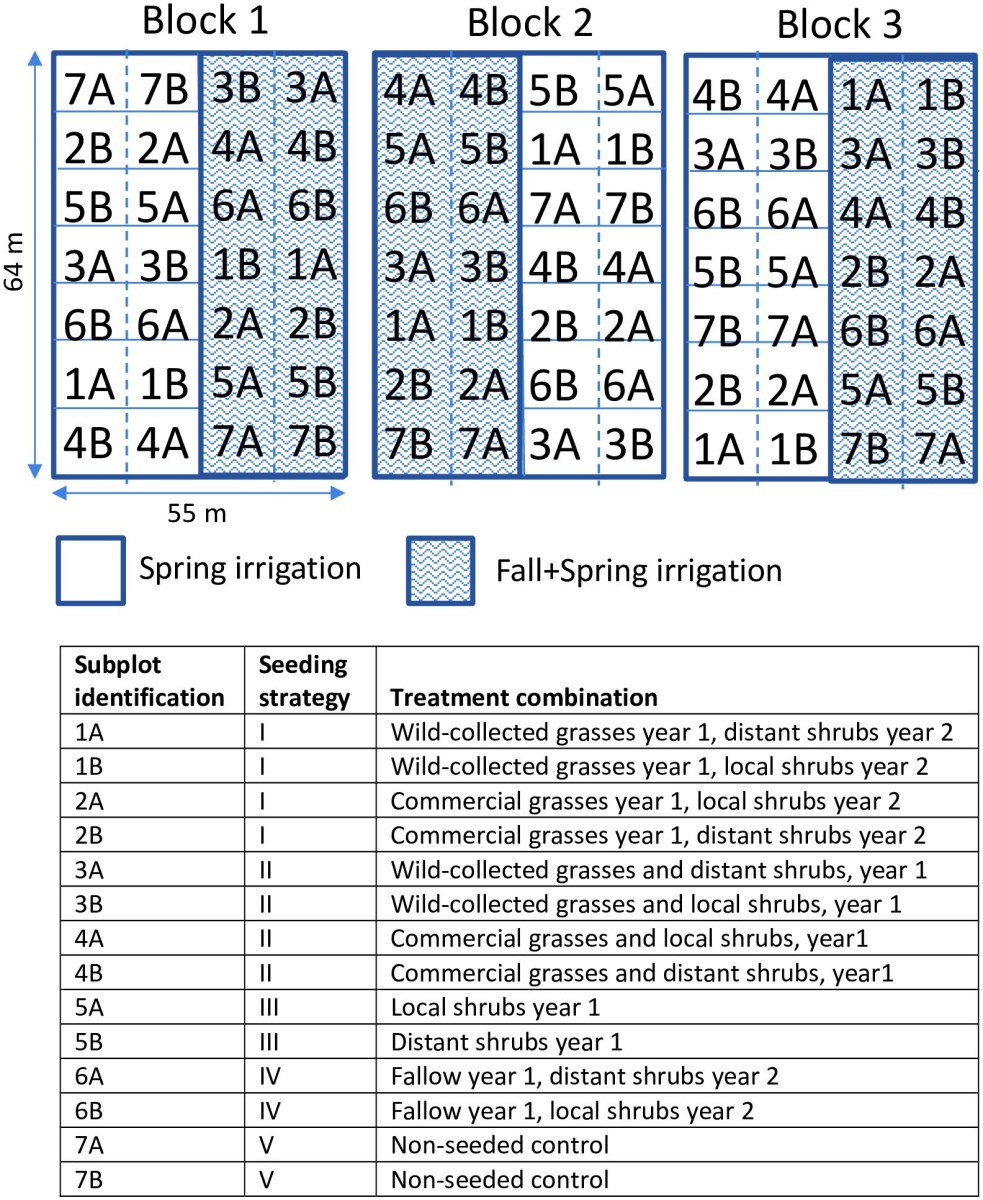
Actual experimental layout schematic at the South Field, illustrating the experimental design used in this study. Half of each block (a 64 m x 27.4 m sub-block) received the same irrigation treatment. Within each sub-block, 7 plots (e.g., 1A and 1B together) were randomly arranged. Plots were then divided into subplots (e.g., 1A, 1B), each randomly assigned with a different origin of shrub seed (local or distant), except for plot 7 which was unseeded (see table above, which identifies each subplot treatment and the corresponding seeding strategy). Though it was unseeded, Plot 7 was also divided into subplots to keep sampling effort consistent among treatments. See Methods for details.

We varied water application, seed source, and seeding strategy (manipulating functional diversity and order of planting) in a split-split plot randomized complete block experimental design. We used five seeding strategies (Table 1) to examine the effects of functional diversity on restoration outcomes: (I) sow grasses in the first year, then shrubs in the second year, (II) sow grasses and shrubs simultaneously in the first year, (III) sow only shrubs in the first year, (IV) sow nothing in the first year, then sow only shrubs in the second year, or (V) sow nothing either year (unseeded control). Broadleaf herbicides were applied in the first year in strategies I and IV, where they could be used to reduce weeds without injuring seeded shrubs, and mowing (described below) was used to reduce weed competition in all but the unseeded control. We also varied the seed source of shrubs and grasses in all strategies. For shrubs, we used two wild-collected seed source origins, one more local (hereafter, “local”) and the other more distant (hereafter, “distant”) from the study site (see details below). For grasses, we used wild-collected seeds from similar habitat types and widely-available commercial seed sources (hereafter, “wild-collected” and “commercial”). Shrub and grass seed origin were fully crossed with seeding strategy, creating 12 unique seeding treatment combinations in 9.1 m x 13.7 m subplots, in addition to unseeded controls (Table 1 and Fig 2).

**Table 1:** Restoration treatments and weed control methods.

Treatments	Seeding strategy				
	I	II	III	IV	V (control)
**Grass in year 1**	yes	yes	no	no	no
**Shrub seeding year**	2	1	1	2	none
**Grass seed origin**	wild-collected / commercial	wild-collected / commercial	--	--	--
**Shrub seed origin**	local / distant	local / distant	local / distant	local / distant	--
**Irrigation regime**	spring / fall+spring	spring / fall+spring	spring / fall+spring	spring / fall+spring	spring / fall+spring
**Weed control**	herbicide+mowing	mowing	mowing	herbicide+mowing	none

Restoration treatments followed five seeding strategies (I-V) that varied in timing of planting and the presence of different functional groups. Seed origins also varied in strategies I-IV, and two different irrigation treatments were applied in all strategies. Forward slash (/) indicates that the listed levels were analyzed separately within the given strategy. Plus signs (+) indicate that treatments were applied together within the given strategy, and dashes (—) indicate when factors were not included in a seeding strategy. Weed control methods are also indicated.

We manipulated the amount and timing of irrigation in two irrigation treatments: (1) spring irrigation only (hereafter, “Spring”), and (2) fall plus spring irrigation (hereafter, “Fall+Spring”). Irrigation was adjusted according to ambient rainfall and capped at a maximum of 229 mm (9 in) of total annual water addition, with the goal of not overly stimulating weed productivity, based on results from previous work [26]. Similarly, we constrained our irrigation treatments to fall and spring, which are coincident with the most favorable growing conditions in this region. We did not continue to irrigate in the summer months, because in our previous work, we observed that summer irrigation is very beneficial for weedy species, while native species tend to be dormant during these months. Irrigation was applied for two years to facilitate seedling establishment. Because establishment was near zero in a non-irrigated treatment in our previous experiment [26], a non-irrigated treatment was not included here. Seeding treatments were arranged into a 55 m x 64 m block of 28 subplots, with half of each block (a sub-block) receiving one of the two irrigation treatments (Spring or Fall+Spring; Fig 2). Irrigation treatments were randomly assigned to sub-blocks. This design was replicated in three blocks at each of two sites (Fig 2).

The species selected for this experiment occur within the region and at nearby sites (S3 Appendix). Shrub species were *Artemisia tridentata* ssp. *wyomingensis* (Wyoming big sagebrush), *Atriplex canescens* (fourwing saltbush), *Ericameria nauseosa* (rubber rabbitbrush), *Sarcobatus vermiculatus* (black greasewood), and *Atriplex torreyi* (Torrey’s saltbush). Grass species were *Achnatherum hymenoides* (Roem. & Schult.) Barkworth (Indian ricegrass), *Sporobolus airoides* (Torr.) Torr. (alkali sacaton), *Elymus elymoides* (Raf.) Swezey (bottlebrush squirreltail), and *Leymus cinereus* (Scribn. & Merr.) Á. Löve (basin wildrye). With the exception of *S*. *airoides*, for which a wild-collection was not available, two sources of seed were obtained for each grass species: a wild-collected source originating from collections as near the planting sites as possible (“wild-collected”), and a commercial source representing commercially-available seeds originating from non-local locations (“commercial”). Because of the single seed source, *Sporobolus airoides* results were removed from analyses of seed origin. Seed sources for shrubs were all wild-collected, as these species are not typically farmed and there are few to no native shrub cultivars developed. Shrubs were sourced from multiple locations, either more distant from the site (“distant,” often lacking precise collection information) or more local to the site (“local,” collected in the region surrounding our experiment). All seed was purchased from Comstock Seed (Gardnerville, NV, USA, S3 Appendix).

### Experimental management and seeding

Prior to planting, we prepared sites using a combination of chemical (glyphosate application in Mar. and Apr. at both sites, and Oct. 2014 at the North Field to target remnant alfalfa) and mechanical treatments (mowing at both sites in Sept. 2014, and spot-treatment with a handheld propane torch at the South Field in Oct. 2014) to remove existing weeds and alfalfa. Seedbed preparation consisted of rototilling to a depth of 10 cm followed immediately by a ring roller cultipacker to firm the soil. First year seeding occurred Oct. 7–10, 2014, and second year seeding between Sept. 29-Oct. 1, 2015. Seeds were sown at a depth of 1.3 cm, within the range of recommended rates [42–44] (S3 Appendix), using a Truax seed drill, followed by press wheels. Grass species were mixed together and seeded in the entire 9.1 m x 27.4 m plot (Fig 2), and shrubs were seeded within each 9.1 m x 13.7 m subplot, with each species planted alone in 1.6 m wide by 9.1 m long single-species strips separated by one 1.6 m wide unseeded strip.

We used chemical and mechanical treatments to reduce weed densities (Table 1). In the first growing season, broadleaf herbicides were applied to plots assigned to seeding strategies I and IV at a rate of 2.3 L AI/ha Weedmaster (2,4-D + dicamba) at the South Field once in May 2015, and 2.3 L AI/ha Weedmaster at both sites once in June 2015; no herbicide was applied to the North Field in May because seeded grasses were too small to survive application. Repeated mechanical mowing (to a 15.2 cm height) was used to control weeds at both sites in June and July 2015 in strategies I-IV. After the second year seeding in fall 2015, weed control was limited to mowing to prevent seed-set, and mowing occurred at both sites in July 2016. No weed control took place on control plots (strategy V) after seeding began in October 2014.

Irrigation was applied using sprinklers mounted on 0.9 m risers, with a distance of 9.1 m between sprinkler heads. In the first year, the Fall+Spring irrigation treatment plots received 51 mm in the fall (2014) and 102 mm in the spring (2015), while the Spring plots received the spring irrigation only (i.e., 102 mm) (Fig 1). In spring 2015, ambient precipitation was above average during the period of April–June 2015 (289% of average), thus spring irrigation was terminated after the 102 mm had been applied. In the second year, the Fall+Spring irrigation treatment plots received 102 mm in the fall (2015) and 13 mm in the spring (2016), while the Spring plots received the spring irrigation only (i.e., 13 mm). Application of 2016 spring irrigation was reduced in volume due to large-sized and very dense weed populations (see below) and high ambient rainfall for the period of April–June (183% of average).

### Data collection

We monitored densities of seeded grasses in December 2014 (no shrubs emerged at this time), and seeded species and weeds in May 2016 and May 2017, either one month (May 2016) or 13 months (May 2017) after the last irrigation event. At all sampling dates, emergent shrubs were monitored by species; grasses were not identified by species until 2017. Grasses and shrubs were assessed in 1 x 1 m quadrats, while weeds were assessed in a randomly selected 25 x 25 cm corner of the quadrat. In cases of very high weed densities, we used a reduced 10 x 10 cm sampling size to improve accuracy of counts. In December 2014, we monitored seeded grasses in three quadrats at random locations in each subplot in each field. In 2016, we randomly placed three quadrats in each of the five monospecific shrub strips in each subplot in the South field, and at least one quadrat per shrub strip in the North field; sampling intensity differed between the two sites due to very low seeding establishment in the North field and limitations to field crew time. Due to the very high weed presence in 2016, we measured maximum height of weeds. In 2017, three quadrats per strip were sampled in both the North and South Fields, and we included a full census of all shrubs present in each subplot in 2017, in addition to counts in quadrats. For all dates, we scaled density counts up to 1 x 1 m, if necessary, and averaged values across quadrats by subplot prior to analysis.

### Statistical analysis

Density of seeded grasses and shrubs, as well as density and height of weeds, were analyzed using linear mixed models in JMP (v. 13.1.0; SAS Institute Inc., Cary, NC, USA). Significance level was set at α = 0.05, and results with borderline significance (i.e., *P*<0.06) are also discussed. Analyses were performed separately for the South Field and North Field, after initial analysis indicated strong differences between sites. Similarly, sampling date was initially included as a main model factor, but because it showed consistently significant interactions with other factors, we analyzed results separately for each sampling date. All models were built from some or all of the following fixed factors and their interactions: irrigation treatment, seeding strategy, shrub origin, and grass origin. When shrub origin was included in the model, analyses were limited to seeding strategies I-IV; when grass origin was included, analyses were limited to seeding strategies I-II. To test the interaction between grass seed origin and shrub seed origin on restoration outcomes, we ran a model with all four factors (irrigation, seeding strategy, shrub origin, and grass origin) and their interactions. To control for the spatial patterns of variation potentially associated with our experimental design, three random effects were also included in the model structure: block, sub-block (nested within block), and plot (nested within sub-block, block). We were unable to model individual shrub and grass species responses due to uneven emergence or establishment of some species in certain treatments, which resulted in many zero counts and non-normal residuals. Thus, models were constructed with functional group response variables (e.g., density of all shrubs, regardless of species), but we present species-level data in figures and tables for shrubs in both years and for grasses in 2017. Exact model structures are specified for shrub and grass density, and weed height and density in model results tables (Tables 2 and 3, and Tables A-B in S4 Appendix). Rather than analyze all possible comparisons between the many different treatment combinations at each site (Fig 2), we used planned contrasts for comparisons that answered our research questions when overall model effects were significant. Prior to analysis, 2016 shrub density, weed density, 2016 weed height, and 2017 North Field grass density data were log transformed (log (x+0.025)), and 2014 and 2017 South Field grass density were square root transformed (√(x+0.025)), to meet the assumptions of homogeneity of variances and normal distribution of residuals. Figures and tables show means ± 1 standard error (SE).

**Table 2:** Model results for shrub density in the South Field in 2016.

Source (fixed factors)^a^	Shrub density (plants m^-2^)^b^		
	*df* ^c^	*F*	*P-*value^d^
***(i) 3-factor model*:**			
Irrigation	1,2.084	0.23	0.67
Seeding strategy	3,24	10.34	**<0.001**
Shrub origin	1,28	35.58	**<0.001**
Irrigation x Strategy	3,24	1.93	0.15
Irrigation x Shrub origin	1,28	0.18	0.68
Strategy x Shrub origin	3,28	10.80	**<0.001**
Irrigation x Strategy x Shrub origin	3,28	0.27	0.85
***(ii) 4-factor model***			
Irrigation	1,2	0.02	0.91
Seeding strategy	1,12	29.88	**<0.001**
Shrub origin	1,16	15.49	**0.001**
Grass origin	1,12	4.00	0.07
Irrigation x Strategy	1,12	1.89	0.19
Irrigation x Shrub origin	1,16	0.34	0.57
Irrigation x Grass origin	1,12	5.26	**0.04**
Strategy x Shrub origin	1,16	14.43	**0.002**
Strategy x Grass origin	1,12	0.32	0.58
Grass origin x Shrub origin	1,16	0.36	0.56
Irrigation x Strategy x Shrub origin	1,16	0.33	0.57
Irrigation x Strategy x Grass origin	1,12	2.24	0.16
Irrigation x Shrub origin x Grass origin	1,16	3.23	0.09
Strategy x Shrub origin x Grass origin	1,16	0.07	0.80
Irrigation x Strategy x Shrub origin x Grass origin	1,16	0.08	0.78

Model results for shrub density in the South Field in 2016, shown for the (i) 3-factor model (Model *P*<0.001, Adj. *R*^*2*^ = 0.84, *N* = 72) and (ii) 4-factor model (Model *P* = 0.002, Adj. *R*^*2*^ = 0.73, *N* = 48). Too few shrubs were encountered in the North Field for analysis. See Methods for details.

^a^ Fixed factors (and levels) included in the 3-factor model: Irrigation treatment (Spring, Fall+Spring), seeding strategy (I, II, III, IV), and shrub origin (local, distant). The 4-factor model added grass origin (wild-collected, commercial) and included only seeding strategy levels I and II (i.e., the treatments where grasses were seeded). Random effects of block, sub-block (nested within block), and plot (nested within sub-block, block) were also included in models but significance of these effects are not shown.

^b^ Shrub density was log transformed prior to analysis.

^c^ Degrees of freedom (*df*), indicated as numerator, denominator *df*.

^d^ Bolded *P*-values indicate significant (*P*<0.05) model effects.

**Table 3:** Model results for grass density in 2016 and 2017 at both sites.

Source (fixed factors)^a^	Grass density (plants m^-2^)^b^		
	*df* ^d^	*F*	*P-*value^e^
***(i) South Field– 2016***			
Irrigation	1,2	0.81	0.46
Seeding strategy	1,12	16.38	**0.002**
Grass origin	1,12	4.03	0.07
Irrigation x Strategy	1,12	4.21	**0.06**^**t**^
Irrigation x Grass origin	1,12	4.48	**0.06**^**t**^
Strategy x Grass origin	1,12	1.56	0.24
Irrigation x Strategy x Grass origin	1,12	2.41	0.15
***(ii) North Field– 2016***			
Irrigation	1,2	15.25	**0.06**^**t**^
Seeding strategy	1,12	25.56	**<0.001**
Grass origin	1,12	1.07	0.32
Irrigation x Strategy	1,12	10.71	**0.007**
Irrigation x Grass origin	1,12	6.37	**0.03**
Strategy x Grass origin	1,12	5.23	**0.04**
Irrigation x Strategy x Grass origin	1,12	0.35	0.57
***(iii) South Field– 2017***			
Irrigation	1,1.98	5.56	0.14
Grass origin	1,16.54	0.56	0.47
Shrub origin	1,19.59	13.70	**0.002**
Irrigation x Grass origin	1,16.54	0.78	0.39
Irrigation x Shrub origin	1,19.59	9.62	**0.006**
Grass origin x Shrub origin	1,19.59	2.12	0.16
Irrigation x Grass origin x Shrub origin	1,19.59	2.55	0.13
***(iv) North Field– 2017***			
Irrigation	1,2	38.50	**0.03**
Grass origin	1,16	26.62	**<0.001**
Shrub origin	1,20	2.76	0.11
Irrigation x Grass origin	1,16	2.07	0.17
Irrigation x Shrub origin	1,20	0.03	0.86
Grass origin x Shrub origin	1,20	2.67	0.12
Irrigation x Grass origin x Shrub origin	1,20	2.06	0.17

Model results for grass density in 2016 and 2017 at the (i) South Field– 2016 (Model *P* = 0.02, Adj. *R*^*2*^ = 0.48, *N* = 48), (ii) North Field– 2016 (Model *P* = 0.003, Adj. *R*^*2*^ = 0.70, *N* = 48), (iii) South Field– 2017 (Model *P* = 0.02, Adj. *R*^*2*^ = 0.73, *N* = 46), and (iv) North Field– 2017 (Model *P*<0.001, Adj. *R*^*2*^ = 0.49, *N* = 48). See Methods for details. Different factors were included in 2016 and 2017 based on model fit. For the South Field– 2017 model, two outliers were removed to improve model fit.

^a^ Fixed factors (and levels) are as follows: Irrigation treatment (Spring, Fall+Spring), seeding strategy (I, II), grass origin (wild-collected, commercial), and shrub origin (local, distant). Random effects of block, sub-block (nested within block), and plot (nested within sub-block, block) were also included in models but significance of these effects are not shown.

^b^ 2017 South Field grass density was square root transformed and 2017 North Field grass density was log transformed prior to analysis, and the analysis excluded *S*. *airoides*.

^d^ Degrees of freedom (*df*), indicated as numerator, denominator *df*.

^e^ Bolded *P*-values indicate significant (*P*<0.05) and bold^t^ indicates borderline significant (*P*<0.06) model effects.

## Ethics statement

Permission was obtained from the Walker Basin Conservancy to perform this research at the Rafter 7 Ranch, outside of Yerington, Nevada, USA. No protected species were identified, sampled, or collected during the course of this study. Since the time of this study, this land has been transferred to the State of Nevada public lands as part of the Walker River State Recreation Area.

## Results

### Question 1: Does functional diversity help or hinder shrub emergence and establishment?

**Fig 3:**
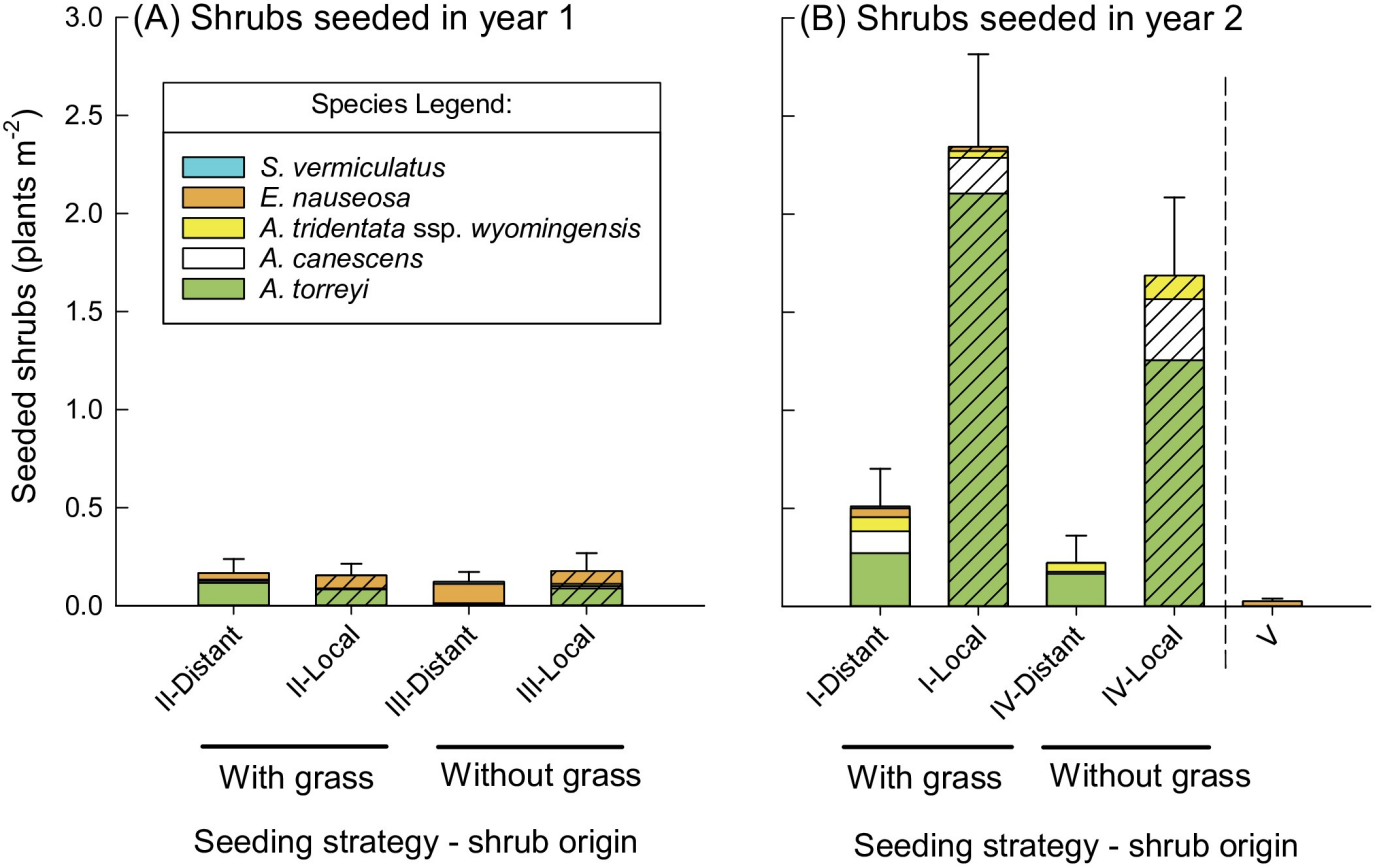
Seeded shrub density in the South Field in May 2016, by seeding strategy and shrub origin. Seeded shrub density (plants m^-2^) in the South Field in May 2016, shown by seeding strategy (I-V; see Table 1) and shrub origin, when shrubs were seeded in year 1 (A) and in year 2 (B), either with or without grasses. “Distant” and “Local” on the X-axis refer to shrub seed origin, which is also indicated by hash marks for local origin, or no pattern for distant origin. Strategy V (unseeded control) is shown in panel (B) for visual comparison of shrub recruitment in areas that did not receive seed addition. There were no differences between irrigation treatments (Table 2(i), S5 Appendix), so results shown here are combined.

In the South Field, functional diversity had either a neutral effect or a facilitative effect on shrub emergence, depending on the restoration seeding strategy and shrub seed origin (seeding strategy*shrub origin interaction, *P*<0.001; Table 2(i); Fig 3). When shrubs were seeded either simultaneously with grasses (strategy II) or without grasses (strategy III) in year one, the presence of grasses did not affect shrub densities of either seed origin, both for local (*P* = 0.9) and distant shrubs (*P* = 0.9) (comparison of strategy II vs. III, Fig 3A). However, when shrubs were seeded in the second year, distant shrub density was 130% higher in the grass-seeded plots relative to plots with shrubs only (*P*<0.05), but there was no effect of seeding grasses on local shrub density (*P* = 0.9) (comparison of strategy I vs. IV, Fig 3B). These results were consistent across irrigation treatments (no significant irrigation main effect or interactions, Table 2(i); S5 Appendix). Emergence of shrubs in unseeded controls was very low (strategy V, Fig 3B).

In seeded plots, *A*. *torreyi* was the most common shrub species in the majority of treatments, followed by *A*. *canescens* when seeded in year 2. *Ericameria nauseosa* was most commonly found in the year 1 seeding, and was the one species that appeared in the unseeded control. *Artemisia tridentata* ssp. *wyomingensis* only emerged when seeded in year 2, and *S*. *vermiculatus* had nearly zero emergence in all treatments and years.

**Fig 4:**
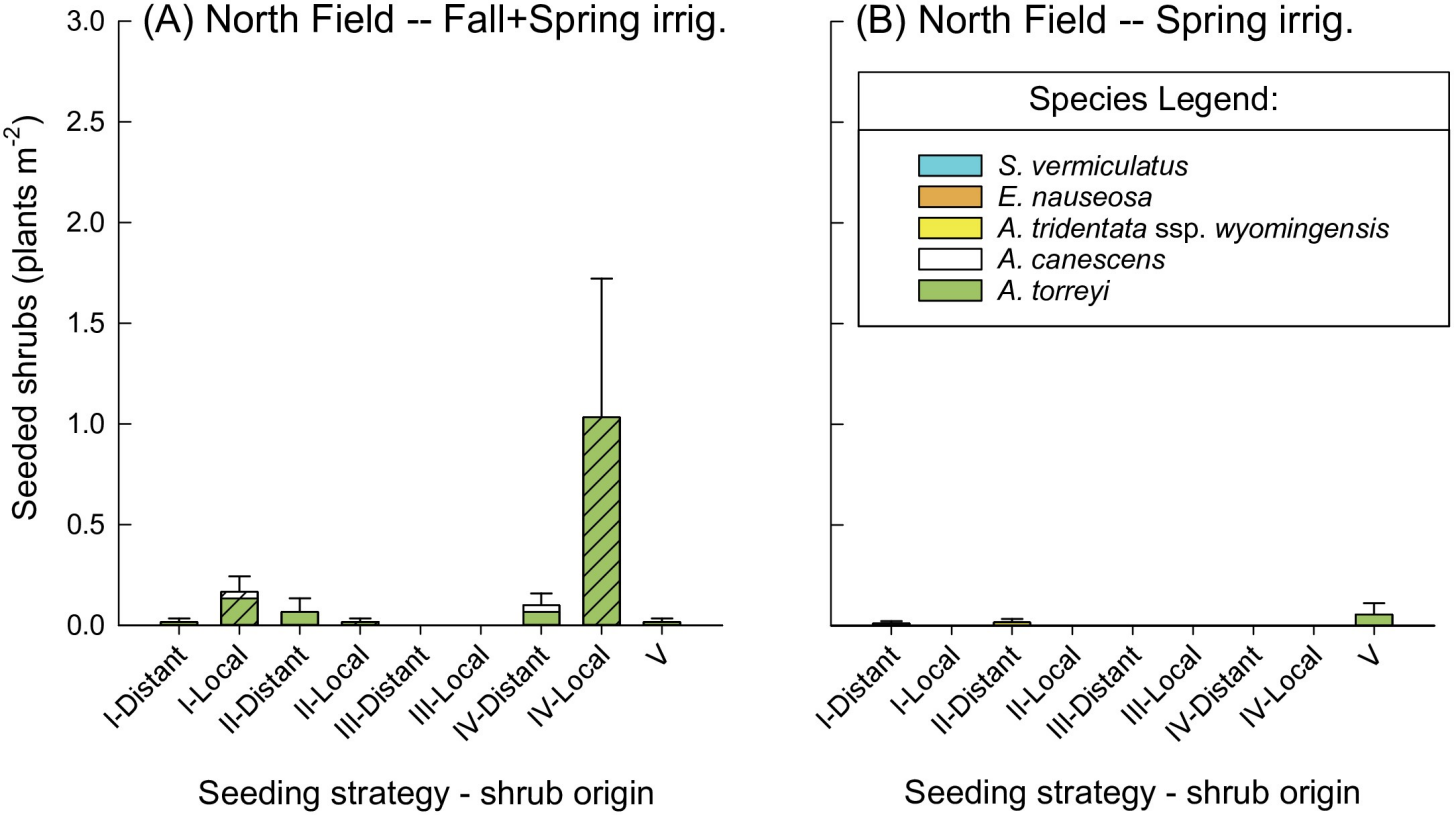
Seeded shrub density in the North Field in May 2016, by irrigation treatment, seeding strategy, and shrub origin. Seeded shrub density (plants m^-2^) in the North Field in May 2016, shown by seeding strategy (I-IV; see Table 1) and shrub origin for the (A) North Field, Fall+Spring irrigation, and (B) North Field, Spring irrigation. “Distant” and “Local” on the X-axis refer to shrub seed origin, which is also indicated by hash marks for local origin, or no pattern for distant origin. Strategy V (unseeded control) is shown in both panels for visual comparison of shrub recruitment in areas that did not receive seed addition.

In the North Field, shrub emergence was extremely low, and we were unable to fit a model of shrub density in 2016, even when treatments with zero values were removed from models. Given this stipulation, qualitatively, shrub emergence in the North Field appeared to be either unaffected by grasses when seeded in year 1 (strategy II vs III), or negatively affected by competition with grasses when seeded in year 2 (strategy I vs. IV) for the Fall+Spring irrigation treatment (Fig 4). Seeding of local shrubs without grasses (strategy IV) in the Fall+Spring irrigation treatment was the only treatment that had appreciable shrub emergence (Fig 4). Successfully emerging shrub species were limited almost entirely to *A*. *torreyi*, with small numbers of *A*. *canescens*.

At both sites, weeds were very dense in 2016 (on average, 469 ± 44 and 662 ± 73 plants m-2 in the North and South Fields, respectively; Fig. A in S4 Appendix), and weeds were substantially taller in the North Field (on average, 25 ± 1 vs. 12 ± 1 cm in the South Field, Fig. B in S4 Appendix). Weed abundance was affected by seeding strategy at both sites (seeding strategy main effect, *P* = 0.02 in the South Field and *P*<0.001 in the North Field; Table A in S4 Appendix), and seeding grasses had either a neutral or facilitative effect on weeds. When shrubs were seeded simultaneously with grasses in year one (strategy II), the presence of grasses did not affect weed abundance (comparison of strategy II vs. III in the South Field (*P* = 0.7) and North Field (*P* = 0.8)). However, when shrubs were seeded a year after grasses and herbicide use (strategy I), the presence of grasses facilitated weeds (comparison of strategy I vs. IV in the South Field (*P* = 0.02) and North Field (*P* = 0.007)). Although weed densities were facilitated by grasses when shrubs were seeded a year after grasses (i.e., strategy I > strategy IV), weed density in this strategy was reduced relative to the control treatment (strategy V) in the North Field (*P*<0.001), and was not significantly different from the control in the South Field (*P* = 0.5). In both the South and North Fields, weed density was also reduced in strategy IV (herbicide followed by shrubs in year two) relative to the control treatment (*P* = 0.01 and *P*<0.001, respectively). The control treatment (strategy V) did not differ significantly from strategies II (*P* = 0.9 and *P* = 0.5) or III (*P* = 0.7 and *P* = 0.4) in the South and North Fields, respectively. Weed densities were not influenced by grass or shrub seed sources, nor were densities impacted by irrigation treatment; we initially included these factors in our analysis, but they were consistently insignificant and resulted in poor model fit.

In 2016, weed height was significantly influenced by grass seeding in the North Field (seeding strategy, *P*<0.001; Table A) but not the South Field (Fig. B in S4 Appendix). Unlike weed density, the presence of grasses reduced weed height in the North Field, both when shrubs were seeded simultaneously with grasses (strategy II vs. III, *P* = 0.005) and after grasses (strategy I vs. IV, *P* = 0.01). Weed height in the control treatment (strategy V) was significantly lower than when shrubs were seeded in year two (strategy IV, *P* = 0.003), but did not differ from the other strategies. In the South Field, none of our experimental factors had an effect on weed height (all *P* >0.05) due to very little variation in the maximum height of weeds across all treatments.

By 2017, after irrigation ceased, weed abundance declined dramatically at both sites, resulting in densities that were approximately 11 times lower in the South Field and 4 times lower in the North Field than in 2016 (Fig. C in S4 Appendix). Weed densities were significantly affected by irrigation legacies in 2017 in the North Field (see Question #3 section below), but there were no other significant effects of seeding treatments on weeds in 2017 (Table B in S4 Appendix).

From 2016 to 2017, shrub density values declined markedly in the South Field, averaging only 3.9 remaining shrubs per 125 m^2^ subplot (Table 4). Already low in 2016, shrub densities declined to near zero in the North Field by 2017. Due to the paucity of data, we were unable to model shrub results in either field for 2017, but we note some patterns we observed. In the South Field, 76% of the shrubs remaining in 2017 were *E*. *nauseosa*, and most of those (84%) were unseeded volunteers growing outside their own seeded strips (Table 4). There were similar shrub density values in the unseeded control (strategy V) compared to seeded treatments (strategies I-IV) in the South Field.

**Table 4:** Total shrub density (and % volunteer) in the South Field in May 2017.

Treatment	Total census shrub density (plants m^-2^ ± SE)				Volunteer shrubs (% of total ± SE)			
	ERNA^a^	ARTR^a^	ATCA^a^	ATTO^a^	ERNA^a^	ARTR^a^	ATCA^a^	ATTO^a^
***Fall+Spring irrigation***								
Seeding strategy I	0	0	0.006±0.006	0	--	--	0	--
Seeding strategy II	0.002±0.001	0	0	0	100±0	--	--	--
Seeding strategy III	0.049±0.042	0.002±0.002	0	0	74±26	100±0	--	--
Seeding strategy IV	0	0.011±0.011	0	0	--	0	--	--
Seeding strategy V	0.028±0.023	0.003±0.002	0	0	100±0	100±0	--	--
***Spring irrigation***								
Seeding strategy I	0.020±0.014	0.028±0.018	0	0.006±0.006	80±20	0	--	0
Seeding strategy II	0.056±0.036	0.006±0.006	0	0	67±21	12±0	--	--
Seeding strategy III	0.028±0.016	0.011±0.011	0	0	84±16	0	--	--
Seeding strategy IV	0.028±0.016	0.011±0.011	0.011±0.011	0	84±16	0	0	--
Seeding strategy V	0.008±0.005	0	0	0	100±0	--	--	--

Shrub density (plants m^-2^) and fraction volunteer shrubs (%) for each species based on a total census of the South Field in May 2017, shown by irrigation treatment and seeding strategy. Note that the North Field is not included in the table due to lack of remaining shrubs in 2017.

^a^ Species codes are as follows: ERNA (*Ericameria nauseosa*), ARTR (*Artemisia tridentata* ssp. *wyomingensis*), ATCA (*Atriplex canescens*), and ATTO (*Atriplex torreyi*). Note that *Sarcobatus vermiculatus* is not shown because all density values were zero.

### Question 2: Does seed source affect restoration outcomes?

The effect of seed source on grass and shrub performance differed between emergence (2016) and early establishment (2017). In the South Field in 2016, the effect of shrub seed source was dependent on seeding strategy (seeding strategy*shrub origin interaction, *P*<0.001, Table 2(i)), with local shrubs outperforming distant shrubs when seeded in year 2 (*P*<0.001; strategies I and IV, Fig 3B), but not when seeded in year 1 (*P* = 0.7; strategies II and III, Fig 3A). These patterns were the same whether grasses were absent (strategies III and IV) or present (strategies I and II). In the North Field, we again were not able to construct models, but local shrubs appeared to have similarly outperformed distant shrubs when seeded in year 2 (strategies I and IV), based on visual assessment (Fig 4A). By 2017, with extremely low shrub densities, there was no obvious difference in shrub origin at either site (Table 4).

When we ran a four-factor model to ask if there were interactions between seed sources of grasses and shrubs in the South Field and irrigation regimes, we found that shrub response to grass seed source was mediated by irrigation regime (grass origin*irrigation interaction, *P* = 0.04, Table 2(ii)). Specifically, in the Fall+Spring irrigation treatment, shrub density was higher when seeded with commercial relative to wild-collected grasses (*P* = 0.01), while shrub density was not affected by grass origin in the Spring irrigation treatment (*P* = 0.8) (S6 Appendix).

**Fig 5:**
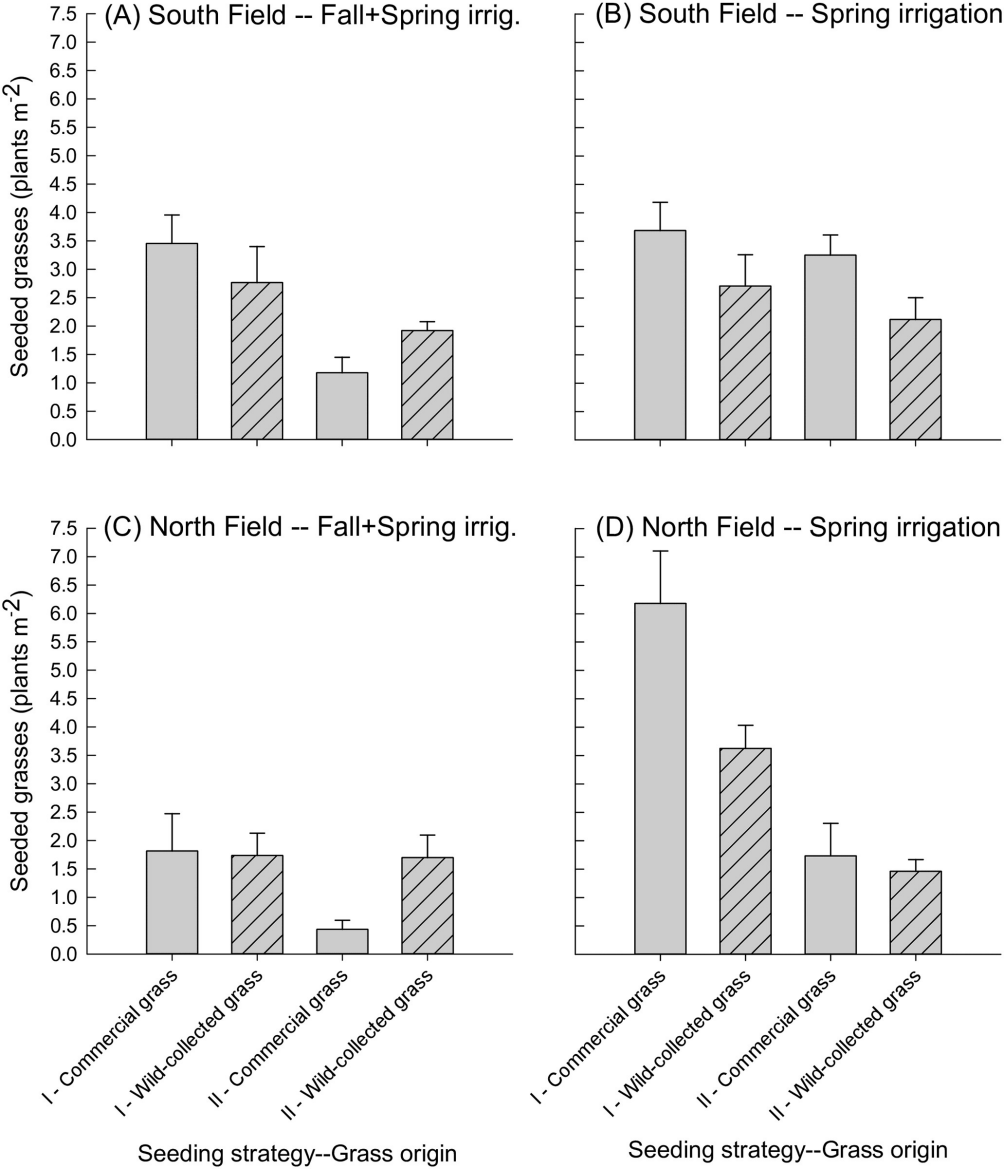
Seeded grasses in May 2016 at both sites, by irrigation treatment, seeding strategy, and grass origin. Seeded grasses (plants m^-2^) in May 2016, shown by seeding strategy (I or II) and grass origin for the (A) South Field, Fall+Spring irrigation, (B) South Field, Spring irrigation, (C) North Field, Fall+Spring irrigation, and (D) North Field, Spring irrigation. *Grass seed origin* is indicated by hash marks for local origin, or no pattern for commercial origin. Seeding strategies are described in Table 1. Note that grass species are not differentiated here because grass seedlings were difficult to identify with certainty at this young stage.

Performance of grasses also differed by seed source and shifted from the seedling stage (2016) to early establishment (2017). In 2016, there were interactions between grass origin and irrigation treatment in both the South and North Fields, and an interaction between grass origin and seeding strategy in the North Fields (Table 3(i) and 3(ii)). In the South Field, contrasts indicated that seed source had no effect on grass densities in the Fall+Spring irrigation (commercial grass in strategies I and II vs. wild-collected grass in strategies I and II; *P* = 0.9; Fig 5A), but in this same comparison, commercial grasses outperformed wild-collected grasses in the Spring irrigation (*P* = 0.01; Fig 5B). In the North Field, there was again no difference among seed sources in Fall+Spring irrigation (commercial grass in strategies I and II vs. wild-collected grass in strategies I and II*; P* = 0.3; Fig 5C), but commercial grasses outperformed wild-collected grasses in the Spring irrigation in strategy I only (commercial grass vs. wild-collected grass in strategy I; *P* = 0.04; Fig 5D). When we ran the four-factor model to test for effects of shrub origin and interactions, these factors were not significant and resulted in poor model fit.

**Fig 6:**
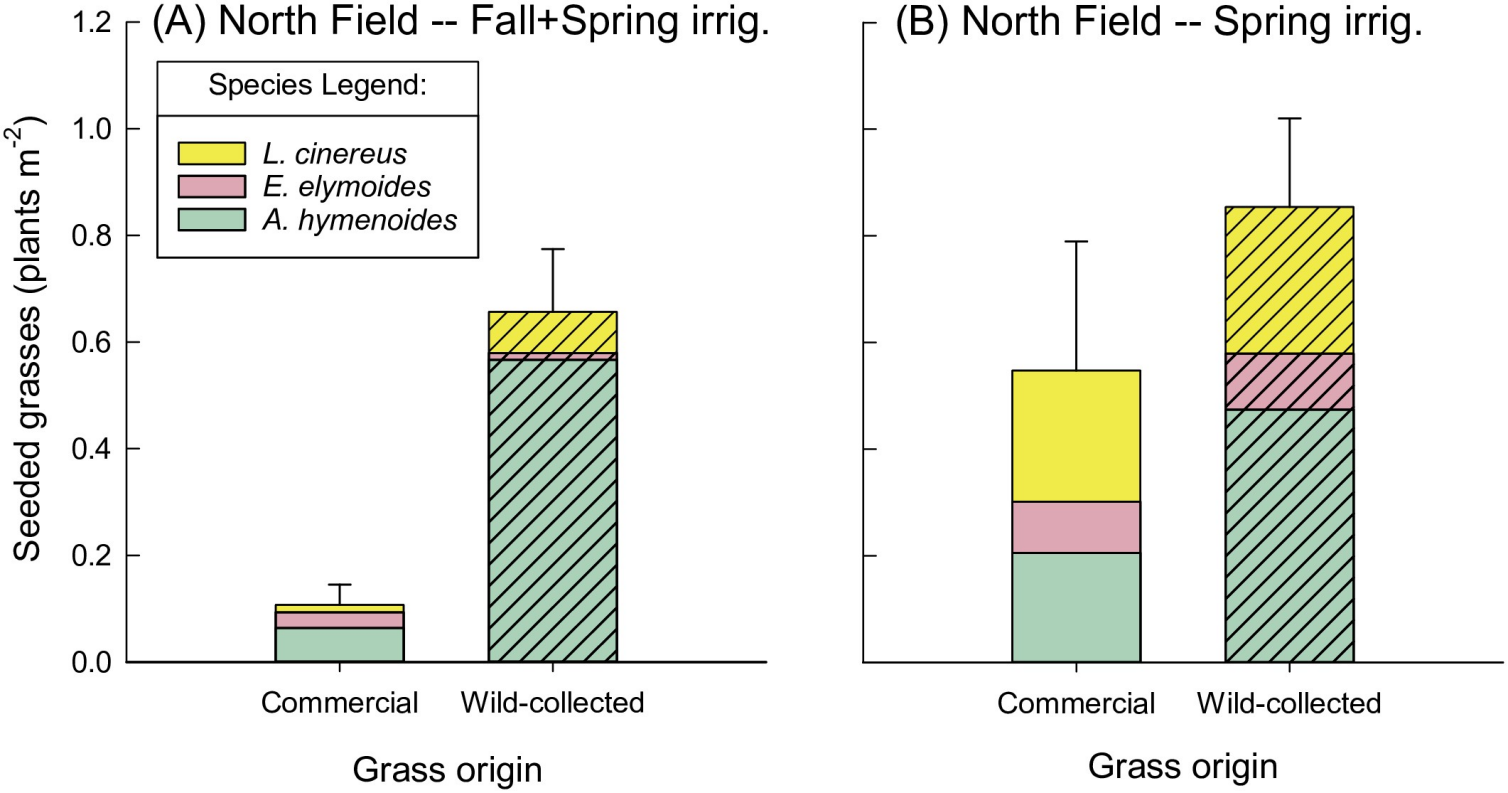
Seeded grasses in the North Field in May 2017, by irrigation treatment and grass origin. Seeded grasses (plants m^-2^) in May 2017, excluding *Sporobolus airoides* (which had only one origin), shown by grass origin for the (A) North Field, Fall+Spring irrigation, and (B) North Field, Spring irrigation. *Grass seed origin* is indicated by hash marks for wild-collected origin, or no pattern for commercial origin. Densities of *S*. *airoides* averaged 1.40±0.98 and 0.66±0.27 plants m^-2^ in the Fall+Spring and Spring irrigation treatments, respectively.

**Fig 7:**
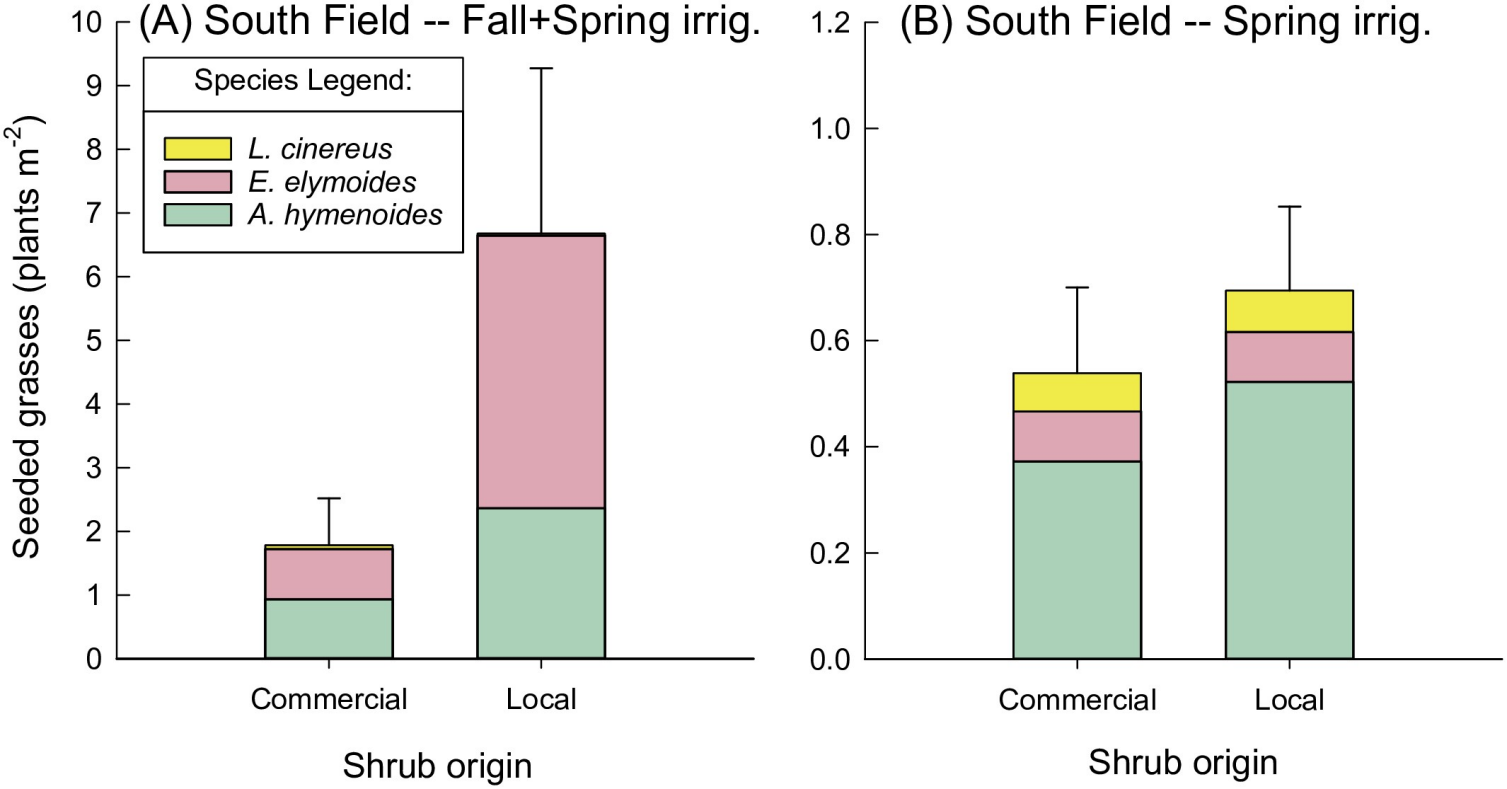
Seeded grasses in the South Field in May 2017, by irrigation treatment and shrub origin. Seeded grasses (plants m^-2^) in May 2017, excluding *Sporobolus airoides* (which had only one origin), shown by shrub origin for the (A) South Field, Fall+Spring irrigation, and (B) South Field, Spring irrigation. Note the larger scale in panel (A) vs. (B). Densities of *S*. *airoides* averaged 0.07±0.02 and 0.52±0.13 plants m^-2^ in the Fall+Spring and Spring irrigation, respectively.

By 2017, any advantages that commercial grasses had over wild-collected grasses in 2016 were lost or reversed (Figs 6 and 7). Irrespective of irrigation effects (explained in the next section), grass density was not affected by grass seed source in the South Field (Table 3(iii)). In the North Field, wild-collected grass density was higher than commercial grass density, averaged across irrigation treatments (grass origin main effect, *P*<0.001, Table 3(iv); Fig 6A and 6B). This appeared to be especially true for *A*. *hymenoides*, which was the most successful species of the three locally-sourced grasses.

In the South Field, though grasses of different origins were not different, overall grass density varied with shrub origin and irrigation (shrub origin*irrigation interaction (*P* = 0.006; Table 3(iii)), with higher grass density when growing with local relative to distant shrubs in the Fall+Spring irrigation (*P*<0.001; Fig 7A), but not in the Spring irrigation (*P* = 0.7; Fig 7B). For both grass and shrub density, there were no significant interactions between grass seed source and shrub seed source, at either site, in either 2016 or 2017.

### Question 3: How does resource availability affect restoration outcomes?

Plant response to irrigation regime was highly context dependent, with effects of irrigation varying by plant functional group and site. Shrub densities were unaffected by irrigation in the South Field in 2016 (Table 2(i), S5 Appendix), but though models could not be run, shrubs appeared to emerge better with Fall+Spring irrigation than with Spring irrigation only in the North Field (Fig 4A vs. 4B). By 2017, shrub densities were almost zero in the North Field, regardless of irrigation treatment, while shrubs appeared to perform better in Spring relative to Fall+Spring irrigation treatment in the South Field, based on visual assessment (Table 4).

**Fig 8:**
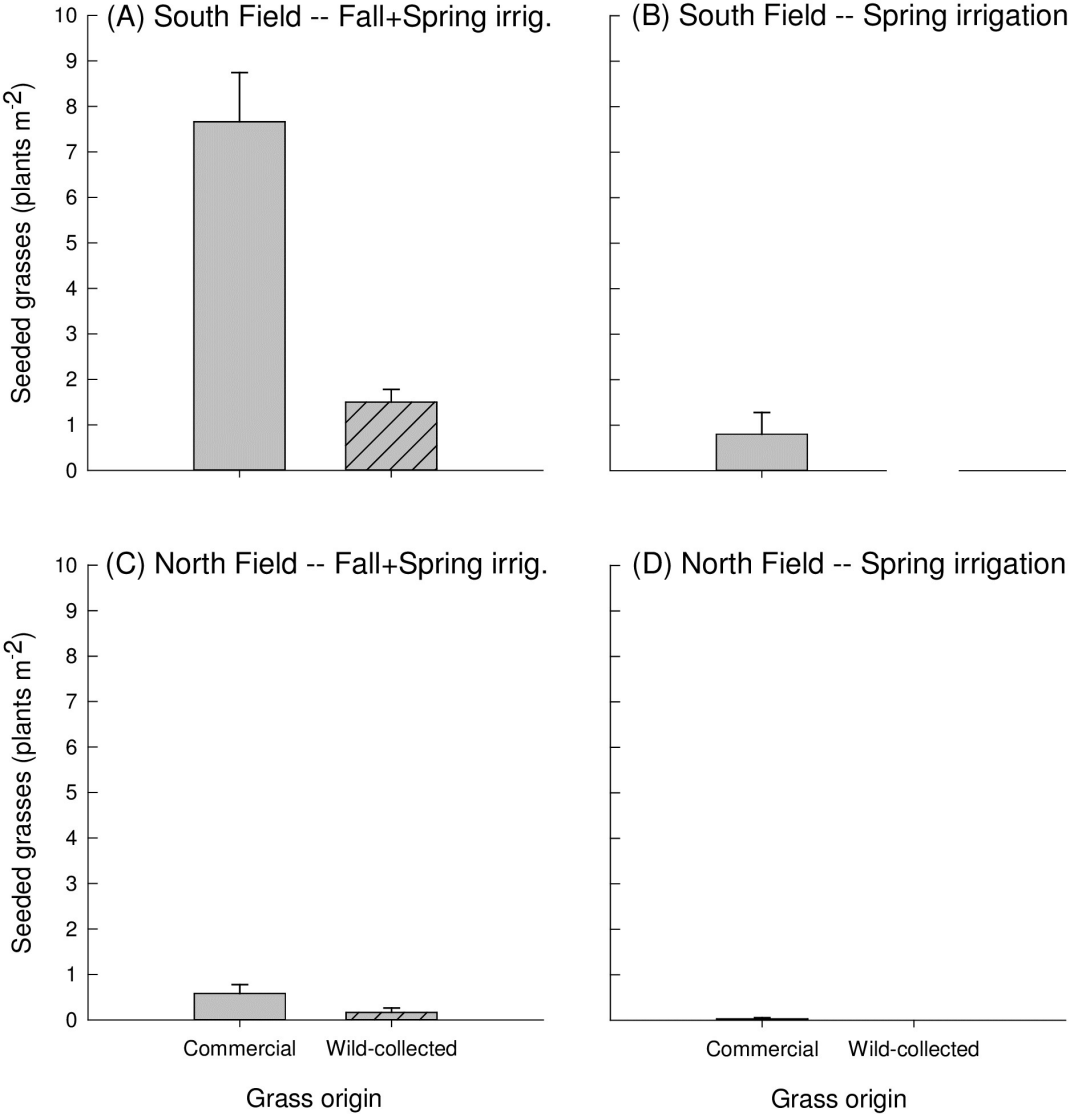
Seeded grasses in the South Field in December 2014, by irrigation treatment and grass origin. Seeded grasses (plants m-2) in December 2014, shown by grass origin for the (A) South Field, Fall+Spring irrigation, (B) South Field, Spring irrigation, (C) North Field, Fall+Spring irrigation, and (D) North Field, Spring irrigation. Grass seed origin is indicated by hash marks for local origin, or no pattern for commercial origin.

In the South Field, density of grasses in December of 2014 was higher in the Fall-Spring irrigation treatment relative to Spring (irrigation main effect, *P* = 0.02, Table 5, Fig 8). There was low grass density in both irrigation treatments in the North Field in December 2014, and we were unable to fit a model to this data. Further, in the South Field, there was a significant interaction between irrigation treatment and grass origin, with emergence higher for commercial grasses relative to wild-collected grasses within the Fall-Spring treatment (*P*<0.001, Table 5, Fig 8).

**Table 5:** Model results for grass density in 2014 in the South Field.

Source (fixed factors)^a^	Grass density (plants m^-2^)^b^		
	*df* ^c^	*F*	*P-*value^d^
***South Field– 2014***			
Irrigation	1,2.02	40.09	**0.02**
Grass origin	1,15.95	61.86	**<0.001**
Irrigation x Grass origin	1,15.95	30.18	**<0.001**

Model results for grass density in 2014 at the South Field (Model *P*<0.001, Adj. *R*^*2*^ = 0.86, *N* = 47). One outlier was removed to improve model fit. Seeding strategy was not included in the model because the full treatments had not been implemented. Too few grasses were encountered in the North Field for analysis.

^a^ Fixed factors (and levels) are as follows: Irrigation treatment (Spring, Fall+Spring) and grass origin (wild-collected, commercial). Random effects of block, sub-block (nested within block), and plot (nested within sub-block, block) were also included in models but significance of these effects are not shown.

^b^ Grass density was square root transformed prior to analysis.

^c^ Degrees of freedom (*df*), indicated as numerator, denominator *df*.

^d^ Bolded *P*-values indicate significant (*P*<0.05) model effects.

In 2016, there was a significant interaction between irrigation and grass origin in the North Field and a near-significant (*P* = 0.06) interaction at the South Field (Table 3(i) and 3(ii)). Wild-collected grass abundance was not affected by irrigation regime in 2016, while commercial grasses exhibited differential responses by field to irrigation (Fig 5). In the South Field, commercial grasses were not significantly affected by irrigation regime when averaged across seeding strategy (*P* = 0.2; Fig 5A vs. 5B). However, in the North Field, commercial grasses were more abundant in the Spring vs. Fall+Spring irrigation (*P* = 0.005; Fig 5C vs. 5D). By 2017, grass density in the South Field was highest when growing with local shrubs in the Fall+Spring irrigation treatment (Fig 7A). In contrast, in the North Field, grasses were overall more abundant in the Spring vs. the Fall+Spring irrigation (irrigation main effect, *P* = 0.03, Table 3(iv); Fig 6). In terms of species composition, it appeared that *A*. *hymenoides* was present at some level in all treatments in both fields (Figs 6 and 7). *Elymus elymoides* appeared to perform the best in Fall+Spring irrigation in the South Field, particularly when growing with local shrubs. In the North field, *L*. *cinereus* had greater establishment in the Spring irrigation treatment.

Finally, neither weed density nor height were different between the two irrigation treatments in 2016 at either site, but by 2017, weeds were significantly more abundant in the Fall+Spring irrigation treatment in the North Field (irrigation main effect, *P* = 0.01, Table B; Fig. C in S4 Appendix).

## Discussion

Some land use changes are very challenging to reverse, and ecological resilience, or the propensity of vegetation to return to a previous state after intervention, can be a negative ecosystem property if change is desired, as is the case in restoration [45,46]. Agricultural legacies are one such example, as they can be apparent in soil and vegetation characteristics for decades, centuries, or even millennia [7,9,47]. Our results indicate that restoration outcomes in former agricultural fields are highly context dependent, with responses differing by site, seeding strategy, seed source, and irrigation regime. Overall, it was evident that transitioning shrubs from the emergence to establishment phase is a large barrier to success in these systems, and we had much greater success in establishing perennial grasses than shrubs in all treatment combinations, with grass densities (~2.7 plants m^-2^ in the South Field, ~1.6 plants m^-2^ in the North Field) nearing those of surrounding natural areas (~2.0 plants m^-2^; unpublished data) by the end of our study. We also had greater plant overall plant emergence and establishment in our lower-fertility field, relative to the more recently cultivated, higher-fertility field, indicating that a period of fallow between active cultivation and restoration attempts may be valuable in similar systems. Seed source is an important factor known to affect restoration outcomes [12,48,49], and during the emergence phase, there were strong indications that local shrubs performed better than non-local ones, and that fall irrigation was important for local shrub emergence in the higher fertility site. In contrast, the performance of commercial grasses was equivalent or superior to wild-collected grasses during emergence, but in the establishment phase, after irrigation ceased, wild-collections were equivalent or superior to commercial sources. Taken together, fall-irrigation of grass + shrub seedings of local origin would be the treatments most likely to produce the most perennial plants in these dryland old-fields.

Increasingly, the theory of plant facilitation is frequently incorporated into restoration practice [50], yet facilitation as a restoration tool remains inadequately studied [51]. It is important to understand how the nature of plant interactions are affected by restoration treatments and underlying site conditions. In support of the predictions of the stress-gradient hypothesis [29], we observed that apparent interactions between grasses and shrubs shifted from facilitation to competition depending on soil resource availability. Specifically, in our lower fertility site, increasing functional diversity with grass seeding either had a neutral or facilitative effect on shrub emergence. Facilitative effects in this site were not due to grass seeding leading to a reduction in weed densities or heights, as we had hypothesized, and thus facilitation may have been the result of other mechanisms such as amelioration of physical stress, which has been observed in other systems where grasses have been found to facilitate other species [21]. In contrast, functional diversity appeared to have a neutral to competitive effect on shrub emergence in our higher fertility site, where grasses established but overall shrub emergence was low, but especially so when seeded with grasses. In other restoration studies, researchers have found that grasses facilitated shrub establishment [22], while in other cases, grasses reduced shrub performance during restoration [26,52]. Rather than a single outcome across multiple conditions, our results support the idea that species interactions during restoration may be highly context-dependent [53], and that considerations of water and soil resource availability are important.

Our study design also allowed us to test potential interactive effects of shrub and grass seed sources in the context of restoration, but we observed no significant interactions between seed sources of the two plant functional types for density responses in any site or in any year. Additionally, grass seed origin itself also did not affect the direction of the interaction between grasses and shrubs, which is surprising given that many of the traits for which grass cultivars are bred could make them highly competitive [48]. However, shrub seed source did affect plant interactions. Facilitation of shrubs by grasses was mediated by shrub seed source in the South Field, supporting the hypothesis that local adaptation may reduce the likelihood of facilitation [39]. Specifically, we saw that while their performance was lower than that of local shrubs, distant shrubs seeded in year 2 were facilitated by the presence of grasses, while local shrubs were not significantly affected. Non-local plants may experience conditions at a new, unfamiliar site as more stressful than their locally adapted counterparts, and facilitation may be stronger, or it may have a larger positive effect on plants that experience their environment as stressful [54]. Despite this increased facilitation for non-local shrubs, we also found that the local shrubs performed substantially better than distant seed sources, suggesting that it is still advisable to use local shrub seed sources when possible in restoration.

Emergence is a critical life stage, one that is often a significant population bottleneck in arid and semiarid ecosystems [55,56]. Therefore, plant responses to restoration treatments during early life stages are key for attaining successful outcomes in the Great Basin [57]. Practices such as short-term irrigation can increase early survival, and utilizing irrigation infrastructure in old field restoration is one of the opportunities provided by past agricultural disturbance [58]. In our study, the effects of irrigation regime were highly context-dependent, differing by site and functional group, and for grasses, by seed source. Because irrigation in fall can be challenging in ecosystems where water is most readily available for spring and summer crops (like alfalfa), it would be easier for practitioners to use spring-only irrigation. However, we found that when fall irrigation stimulated grass emergence, as in our lower fertility South Field, this resulted in higher grass densities than spring-only irrigation. At this site, a spring-only irritation treatment would have been as effective as fall irrigation for shrub emergence. At the more challenging higher fertility site, fall irrigation was key for stimulating any level of germination and emergence of shrubs, but fall irrigation did not stimulate grass emergence, and grass densities remained lower throughout the entire experiment. It is possible that seeds germinated in response to fall irrigation but failed to emerge, which has been observed in other restoration trials in the northern Great Basin [57]. Finally, while our previous experiment found that irrigation in the summer stimulated large weed responses, it is possible that continuing irrigation and aggressive weed control through the summer months may ultimately increase shrub establishment. Innovating ways to keep emerging shrubs alive through the establishment phase will be of primary importance for reaping any benefits of fall-irrigation in this ecosystem.

In general, weeds are a significant barrier to native re-establishment during restoration [25], and dryland old-fields typically have large weed reserves in the seed bank [59]. The legacy effects of former agriculture were stronger in the North Field, where higher site fertility, and concomitant increased competitive pressure from dense, tall weeds, likely played an instrumental role in the strikingly low shrub emergence followed by almost complete failure in 2017. In this experiment, we did not measure light availability, but other studies have shown that competition for light can be a significant factor influencing early establishment of woody species, even in deserts [60,61]. In more productive conditions, competition for light may prevail [35,36], and in our study, shrub emergence may have been largely suppressed by weed competition, despite our efforts to reduce weed height by mowing. By 2017, after irrigation ceased, weed densities declined at both sites, but remained higher in the North Field (Fig. B in S4 Appendix), possibly contributing to continued shrub attrition. Dominance of weeds can result in low native seedling emergence and survival, even with the addition of irrigation, during restoration of dryland abandoned agricultural areas. Future work focused on reducing weed seed banks may be able to increase establishment success, and mechanistic studies could determine whether ameliorating aboveground competition for light or below-ground competition for soil resources are more important for native plant establishment.

We hoped to use perennial grass seeding as a method for reducing weed pressure. Achieving grass densities that allow for shrub establishment while also resulting in weed suppression is a delicate balancing act, influenced by restoration treatments, such as grass seeding rates, irrigation, and species identity [26,27,62], as well as uncontrolled environmental factors, such as precipitation [28]. Similar to our previous findings [26], we saw that grasses initially suppressed or had a neutral effect on weeds relative to an unseeded control, but that they had neutral or facilitative effects on weeds relative to treatments seeded with shrubs only. As intended, grass densities in this study were lower than those in the previous work (~30 to 75 plants m^-2^ after two years of irrigation); our effort to reduce competitive effects and increase facilitative ones was successful. Reduction of weed density was consistently observed in treatments where herbicide was applied without grass seeding, and in our higher fertility site, the presence of grasses further reduced weed height, and this was the case whether herbicide was applied or not. Our findings suggest that the seeding of grasses can be compatible with shrub emergence during restoration in these ecosystems, and that there may be weed-suppressive benefits of perennial grasses in higher-fertility old fields.

We included multiple species in our seeding mix, in addition to multiple seed sources, and there were several cases where species-specific responses appeared to be responsible for positive restoration outcomes in particular treatments. This is consistent with ideas about links between biodiversity and stabilizing community-level processes, such as the portfolio effect or statistical averaging effect [63,64], which have direct links to restoration success [65]. Only one species, *A*. *hymenoides*, a native grass common in the arid and semiarid western USA and found across a range of soil conditions [66], was present at some level in all treatments. Thus, in our study, most species did not respond to restoration treatments in concert, and the fact that we seeded a diverse mix with a variety of species and functional groups maximized the chance for successful establishment across a variety of conditions.

Finally, our results suggest that a “water it and they will come” approach may lead to shrub establishment over a long-term time frame in some dryland old fields. Natural recruitment appeared to be the largest source of *E*. *nauseosa* shrubs, which accounted for 76% of the established shrubs in the South Field, including in the unseeded, no herbicide control treatment (Table 4). Our findings showing natural recruitment of *E*. *nauseosa* in our irrigated plots are similar to previous research on old fields in this desert ecosystem, where most shrub recruitment originated from surrounding areas and was predominantly *E*. *nauseosa*, with most recruitment in areas with supplemental irrigation, no seeded grasses, and where the soil surface had been disturbed with a rangeland drill [26]. Generally, *E*. *nauseosa* and *A*. *tridentata* do best when seeded on top of disturbed soil [43], and thus these species may establish well from natural sources close to irrigated restoration treatments, if native seed sources are in close proximity and weed pressure is not overly high.

## Conclusions

Restoration in dryland environments is complex and difficult, and in the Great Basin, restoration efforts often result in limited success or outright failure [67]. In our study, we observed some successes, with results highly variable across treatments. We succeeded at the emergence stage for grasses and most shrub species, although shrub emergence was low in the North Field. In most treatments, we also succeeded at grass establishment: at the end of the study, grass densities in the two old fields were comparable to less disturbed surrounding areas which had not been converted to agriculture. Overall, our treatments failed to transition seeded shrubs from emergence to establishment, with the exception that we created conditions conducive to natural shrub recruitment. High rates of seeded shrub attrition in the last year of the study may have been related to water stress, competition from weeds, or from herbivory, and our results indicate that finding methods to alleviate this bottleneck will be key to successful restoration of these systems, especially in higher fertility sites with recent agricultural activity. We caution, however, that while our experiment manipulated many factors simultaneously, a trade-off is that we had only limited levels of each factor (two irrigation treatments, two seed sources, etc.), and thus we had no capacity to detect threshold effects, for example, with more nuanced differences in seed source.

Our findings suggest that the use of local seed sources is warranted, as there were either positive or neutral effects of using more local and wild-collected seeds. In contrast, results from functional diversity and irrigation regime manipulations were highly context-dependent, and indicate that further studies that increase the number of levels of these treatments could help pinpoint under what circumstances these factors help or hinder restoration. The context-dependency of restoration outcomes is increasingly recognized in restoration ecology research [68]. Our results support the concept that a bet-hedging strategy that uses, for example, a variety of seeding strategies and irrigation regimes within an overall restoration plan, is the best way to maximize the chance of positive restoration outcomes in challenging, variable environments [69].

## Supporting information

S1 Appendix Weed composition at the study sites. (PDF)

S2 Appendix Soil characteristics at the study sites. (PDF)

S3 Appendix Species and seeding rates used in the experiment, with location of seed origins. (PDF)

S4 Appendix Results tables and figures for weed abundance and height in 2016 and 2017. (PDF)

S5 Appendix Results figure for shrub density in 2016 in the South Field, shown separately by irrigation regime. (PDF)

S6 Appendix Results figure for shrub density in 2016 in the South Field, as related to the 4-factor model (Table 2(ii)). (PDF)
