# Supplementary material for: Restoring dryland old fields with native shrubs and grasses: Does facilitation and seed source matter?
Source: PLoS One. 2018 Oct 18;13(10):e0205760. doi: 10.1371/journal.pone.0205760 (PMC6193679; doi:10.1371/journal.pone.0205760)
Supplement: S1 Appendix — (PDF) [file pone.0205760.s001.pdf]

S1 Appendix. Weed composition at the study sites.*

	Site	
**Weed species**	**North Field**	**South Field**
Amaranthus spp. (pigweed)		✓
Bromus madritensis (compact brome)		✓
Bromus tectorum (cheatgrass, downy brome)	✓	✓
Chenopodium album (lamb's quarters)	✓	
Chenopodium spp. (goosefoot)	✓	✓
Convolvulus arvensis (field bindweed)	✓	✓
Descurainia sophia (tansy mustard, flixweed)	✓	✓
Erodium cicutarium (redstem filaree, stork's bill)	✓	✓
Hordeum jubatum (foxtail barley)	✓	✓
Kochia scoparia (common kochia)	✓	✓
Lepidium latifolium (perennial pepperweed, tall whitetop)		✓
Malva neglecta (common mallow, cheeseweed)	✓	
Medicago sativa (alfalfa)	✓	✓
Polygonum spp. (knotweed)	✓	
Portulaca spp. (purslane)	✓	
Salsola tragus (prickly Russian thistle)	✓	✓
Sisymbrium altissimum (tall tumblemustard)	✓	✓
Tragopogon spp. (salsify)	✓	

* Indicated as presence or absence during monitoring (May 2017), listed alphabetically by species.
