# Supplementary material for: Restoring dryland old fields with native shrubs and grasses: Does facilitation and seed source matter?
Source: PLoS One. 2018 Oct 18;13(10):e0205760. doi: 10.1371/journal.pone.0205760 (PMC6193679; doi:10.1371/journal.pone.0205760)
Supplement: S2 Appendix — (PDF) [file pone.0205760.s002.pdf]

S2 Appendix. Soil characteristics at the study sites.*

Soil Characteristic	North Field	South Field**
pH	7.2 ± 0.2	7.4 ± 0.04
$EC_{1:2}$ (dS m^{-1})***	0.39 ± 0.11	0.12 ± 0.01
C (%)	1.34 ± 0.11	0.48 ± 0.02
N (%)	0.15 ± 0.01	0.05 ± 0.002
$\delta^{15}N$ (‰)	5.1 ± 0.1	5.5 ± 0.1
C:N	8.8 ± 0.1	9.3 ± 0.1
Nitrification potential rate (mg N kg^{-1} d^{-1})	13.6	1.8 ± 0.5
P (mg P kg^{-1})	129.3 ± 18.6	64.7 ± 2.8
Ca (mg Ca kg^{-1})	2645.2 ± 158.0	1523.1 ± 43.9
Mg (mg Mg kg^{-1})	474.1 ± 41.5	252.7 ± 8.5
K (mg K kg^{-1})	522.3 ± 68.4	296.0 ± 15.1
Na (mg Na kg^{-1})	129.7 ± 14.8	78.3 ± 3.3
ESP (%)***	5.4 ± 0.5	5.8 ± 0.2
SAR***	0.2 ± 0.02	0.2 ± 0.01
Sand (%)	53.9 ± 2.2	78.8 ± 0.9
Silt (%)	29.3 ± 1.8	15.9 ± 0.8
Clay (%)	16.8 ± 1.8	5.4 ± 0.2
Soil texture	Sandy loam	Loamy fine sand

Mean ± SE (*n*=3 for the North Field and *n*=28 for the South Field for all values, except potential nitrification rate where *n*=1 (composite of three subsamples) for the North Field and *n*=14 for the South Field.)

* Methods described in detail in Dunham-Cheatham SM, Freund SM, Uselman SM, Leger EA, Sullivan BW. Persistent agricultural legacy influences plant restoration success in a native salt desert shrubland. Agriculture, Ecosystems, and Environment (*In Review*).

** South Field data from Dunham-Cheatham et al. (*In Review*).

*** Abbreviations: EC = Electrical conductivity; ESP = Exchangeable sodium percentage; SAR = Sodium adsorption ratio.
