# Supplementary material for: Restoring dryland old fields with native shrubs and grasses: Does facilitation and seed source matter?
Source: PLoS One. 2018 Oct 18;13(10):e0205760. doi: 10.1371/journal.pone.0205760 (PMC6193679; doi:10.1371/journal.pone.0205760)
Supplement: S3 Appendix — (PDF) [file pone.0205760.s003.pdf]

S3 Appendix. Species and seeding rates used in the experiment, with location of seed origins. Our seed source was able to provide information on the state of origin for less local origins, and state/county for more local origins.

**Shrubs**	**Distant origin (less local)**	**Local origin (more local)**	**Rate (kg PLS ha^{-1})**
A. tridentata ssp. *wyomingensis*	California	Northwestern NV (Humboldt/Pershing/Washoe Co.)	0.56
A. canescens	Arizona	Lyon/Churchill Co., NV	2.24
E. nauseosa	Nevada	Lyon/Washoe Co., NV	0.056
S. vermiculatus	Nevada	Lyon Co., NV	2.24
A. torreyi	Arizona	North Central NV	2.24

**Grasses**	**Commercial origin**	**Wild collection**	**Rate (kg PLS ha^{-1})**
A. hymenoides	'Nezpar', ID	Mono Co., CA	2.24
S. airoides	VNS[a], UT (commercially grown)	--	0.280
E. elymoides	'Klamath', OR	Mono Co., CA	2.24
L. cinereus	'Trailhead', MT	Lassen Co., CA[b]	2.80

[a]VNS = variety not stated.
[b]Honey Lake Basin.
