# Supplementary material for: Restoring dryland old fields with native shrubs and grasses: Does facilitation and seed source matter?
Source: PLoS One. 2018 Oct 18;13(10):e0205760. doi: 10.1371/journal.pone.0205760 (PMC6193679; doi:10.1371/journal.pone.0205760)
Supplement: S4 Appendix — (PDF) [file pone.0205760.s004.pdf]

S4 Appendix. Results tables and figures for weed abundance and height in 2016 and 2017.

Model results are reported for weed density and height in 2016 in Table A, and weed density in 2017 in Table B. Weed density and height are illustrated as a function of year, site, irrigation treatment, and seeding strategy in Figures A-C. Main results are explained in the text of the Results.

Table A. Model results for weed density and height in 2016 at the (i) South Field (Density: model P=0.02, Adj. R^2=0.61, N=84; Height: model P=0.42) and (ii) North Field (Density: model P<0.001, Adj. R^2=0.86, N=84; Height: model P<0.001, Adj. R^2=0.07, N=84). See Methods for details.

	**Weed density (plants m^{-2})[a]**			**Maximum weed height (cm)[a]**		
**Source (fixed factors)[b]**	***df*** **[c]**	***F***	***P*-value[d]**	***df*** **[c]**	***F***	***P*-value[d]**
(i) South Field						
Seeding strategy	4,32	3.29	**0.02**	NA[e]	NA[e]	NA[e]
(ii) North Field						
Seeding strategy	4,32	30.72	**<0.001**	4,32	6.57	**<0.001**

[a] Weed density and height were log transformed prior to analysis.
[b] Fixed factors (and levels) are as follows: Seeding strategy (I, II, III, IV, V). Random effects of block, sub-block (nested within block), and plot (nested within sub-block, block) were also included in models but significance of these effects are not shown.
[c] Degrees of freedom (*df*), indicated as numerator, denominator *df*.
[d] Bolded *P*-values indicate significant (P<0.05) model effects.
[e] NA[e] = Not Applicable, because weed height in the South Field could not be modeled. See Results text for details.

Table B. Model results for weed density in the North Field in 2017 (Model P=0.05, Adj. R^2=0.76, N=84)[a]. See Methods for details.

**Source (fixed factors)**[c]	**Weed density (plants m^{-2})**[b]		
	df [d]	***F***	***P*-value**[e]
Irrigation	1,4.1	17.08	**0.01**
Seeding strategy	4,28	1.05	0.40
Irrigation x Seeding strategy	4,28	0.56	0.69

[a] Weed density in 2017 could not be modeled in the South Field, as density did not vary significantly with any of the experimental factors.

[b] Weed density in 2017 was log transformed prior to analysis.

[c] Fixed factors (and levels) are as follows: Irrigation treatment (Spring, Fall+Spring) and seeding strategy (I, II, III, IV, V). Random effects of block, sub-block (nested within block), and plot (nested within sub-block, block) were also included in models but significance of these effects are not shown.

[d] Degrees of freedom (*df*), indicated as numerator, denominator *df*.

[e] Bolded *P*-values indicate significant (P<0.05) model effects.

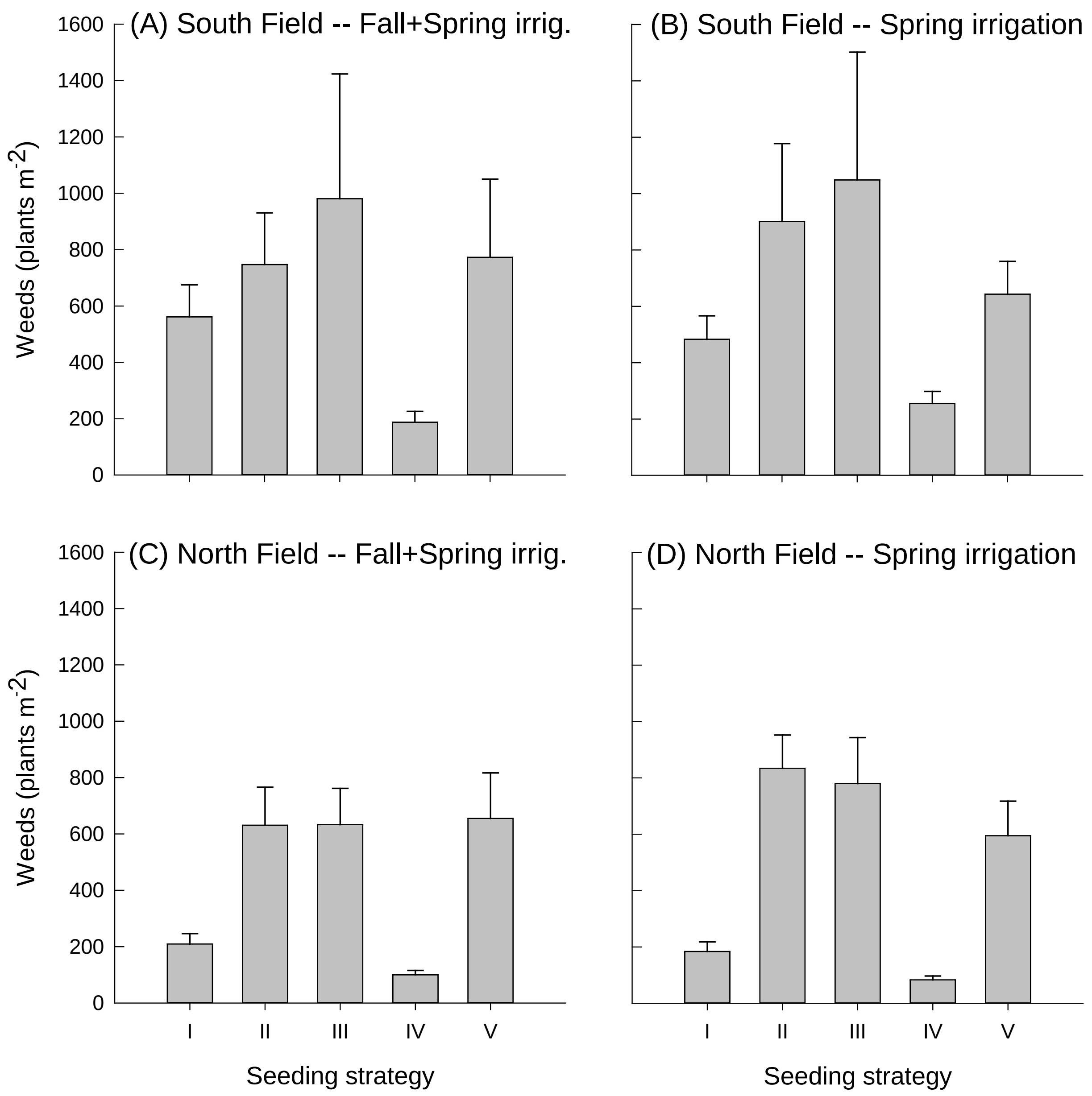

Fig. A. Weed density (plants m^{-2}) in 2016, shown by seeding strategy for the (A) South Field, Fall+Spring irrigation, (B) South Field, Spring irrigation, (C) North Field, Fall+Spring irrigation, and (D) North Field, Spring irrigation.

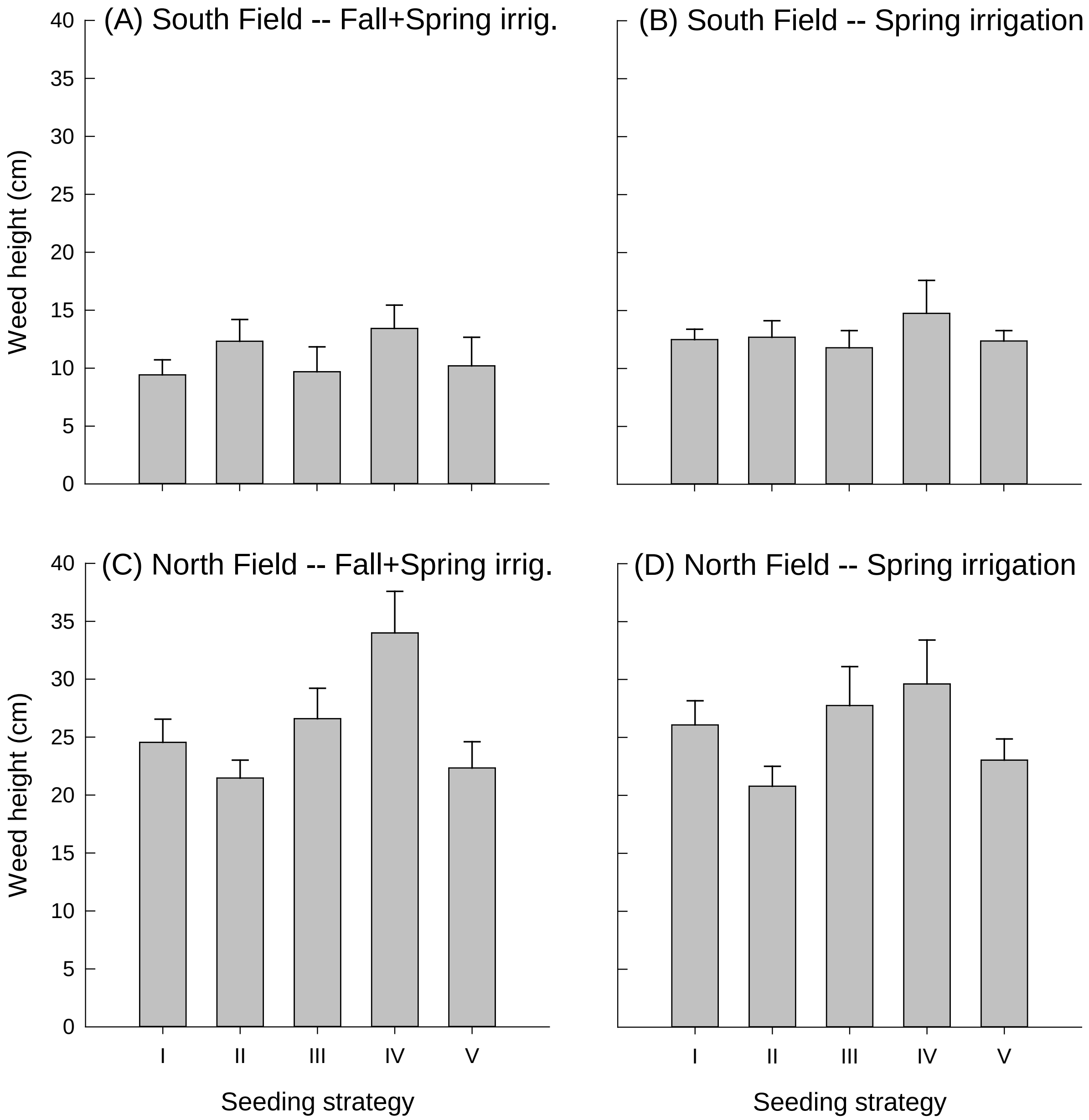

Fig. B. Weed height (cm) in 2016, shown by seeding strategy for the (A) South Field, Fall+Spring irrigation, (B) South Field, Spring irrigation, (C) North Field, Fall+Spring irrigation, and (D) North Field, Spring irrigation.

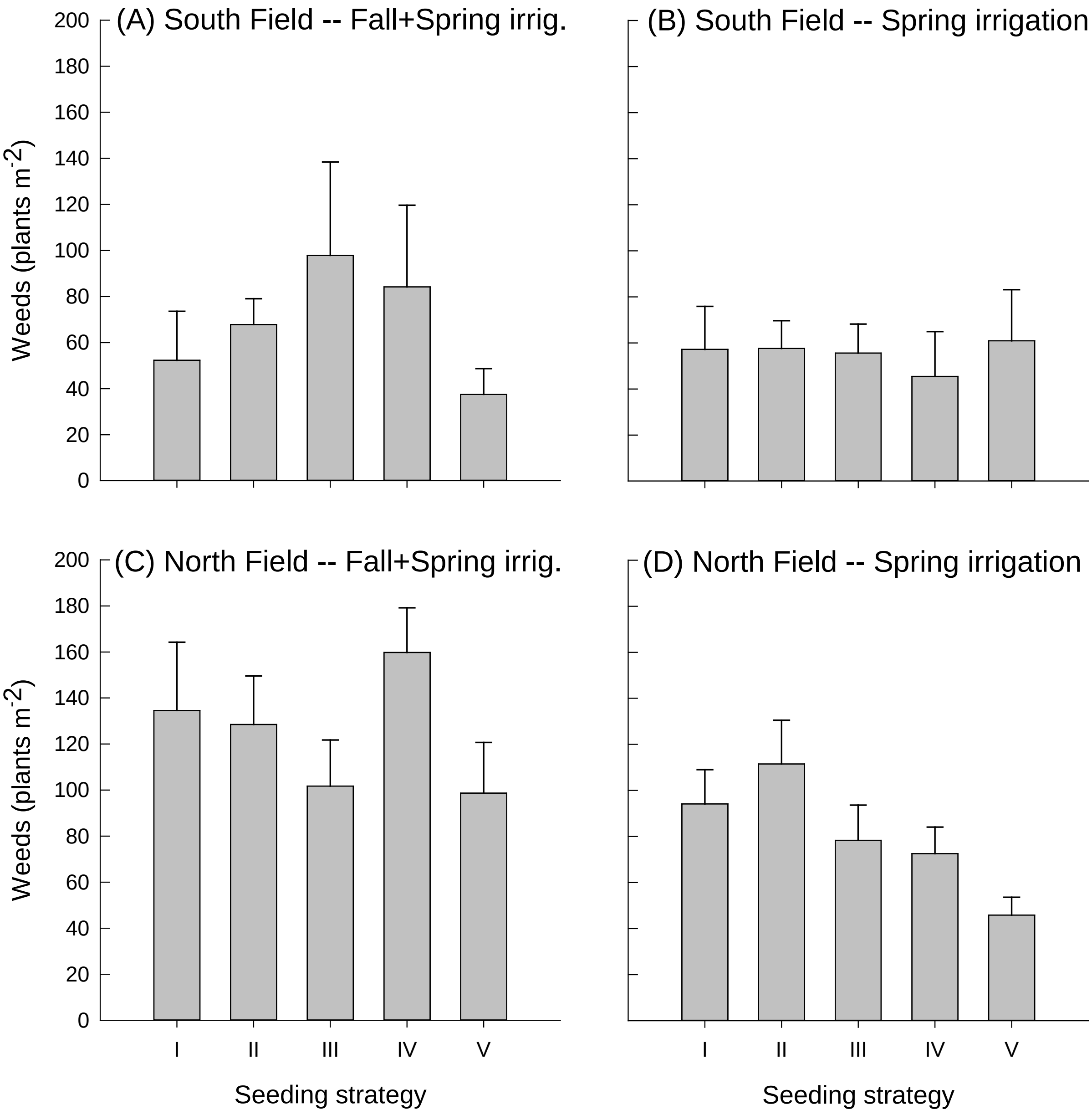

Fig. C. Weed density (plants m^{-2}) in 2017, shown by seeding strategy for the (A) South Field, Fall+Spring irrigation, (B) South Field, Spring irrigation, (C) North Field, Fall+Spring irrigation, and (D) North Field, Spring irrigation.
