# Supplementary material for: Restoring dryland old fields with native shrubs and grasses: Does facilitation and seed source matter?
Source: PLoS One. 2018 Oct 18;13(10):e0205760. doi: 10.1371/journal.pone.0205760 (PMC6193679; doi:10.1371/journal.pone.0205760)

S5 Appendix. Results figure for shrub density in 2016 in the South Field, shown separately by irrigation regime. Seeded shrub density (plants m^{-2}) in the South Field in May 2016, shown by seeding strategy (I-IV; see Table 1) and shrub origin for the (A) South Field, Fall+Spring irrigation, and (B) South Field, Spring irrigation. "Distant" and "Local" on the X-axis refer to shrub seed origin, which is also indicated by hash marks for local origin, or no pattern for distant origin. Strategy V (unseeded control) is shown in both panels for visual comparison of shrub recruitment in areas that did not receive seed addition. There were no significant differences among irrigation treatments in the South Field, but results are shown separately to allow for visual observation of differences among species.

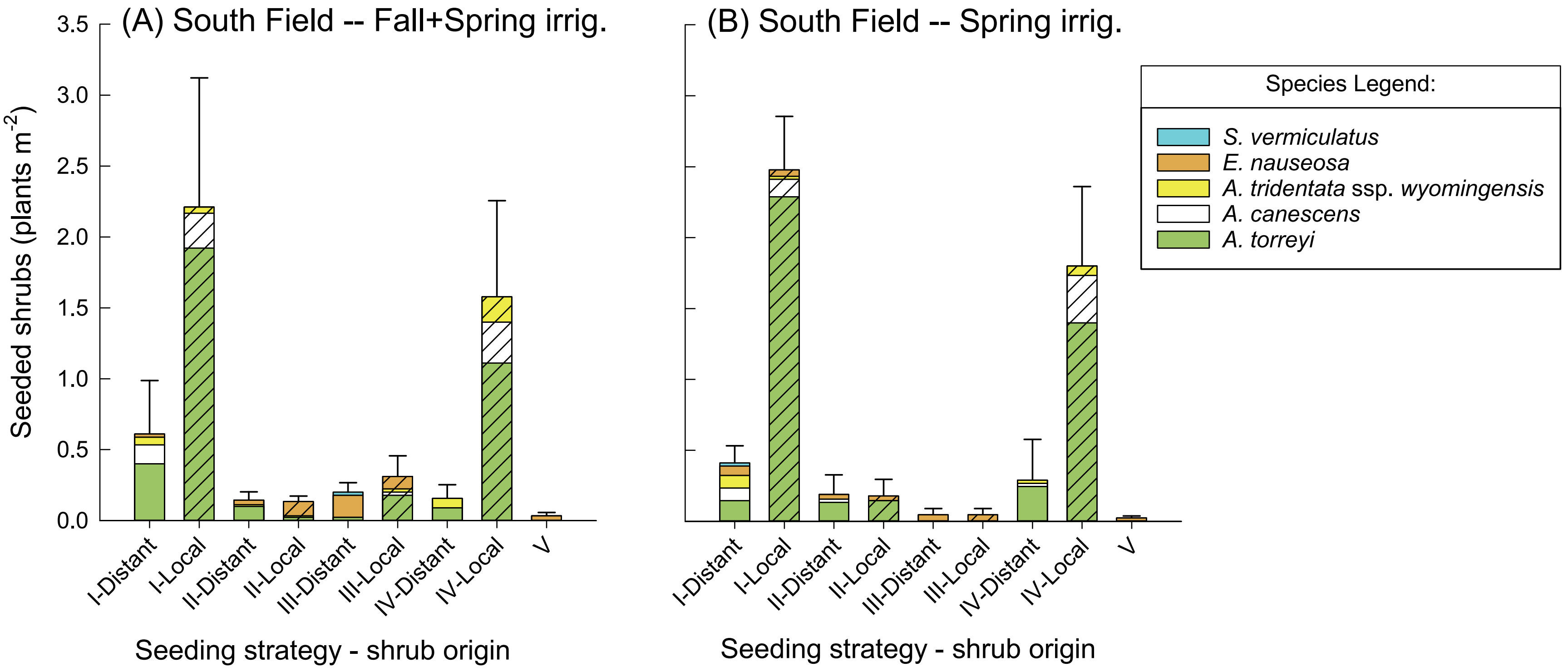

Supplement: S5 Appendix — (PDF) [file pone.0205760.s005.pdf]
