# Supplementary material for: Restoring dryland old fields with native shrubs and grasses: Does facilitation and seed source matter?
Source: PLoS One. 2018 Oct 18;13(10):e0205760. doi: 10.1371/journal.pone.0205760 (PMC6193679; doi:10.1371/journal.pone.0205760)

S6 Appendix. Results figure for shrub density in 2016 in the South Field, as related to the 4-factor model (Table 2(ii)). Seeded shrub density (plants m^{-2}) in May 2016 is shown by seeding strategy (I or II), shrub origin, *and grass origin* for the (A) South Field, Fall+Spring irrigation, and (B) South Field, Spring irrigation. “Distant” and “Local” on the X-axis refer to shrub seed origin, which is also indicated by hash marks for local origin, or no pattern for distant origin. Note that only the South Field is shown here, where the 4-factor model was fit to the data (see Methods for details; Table 1 for descriptions of seeding strategies).

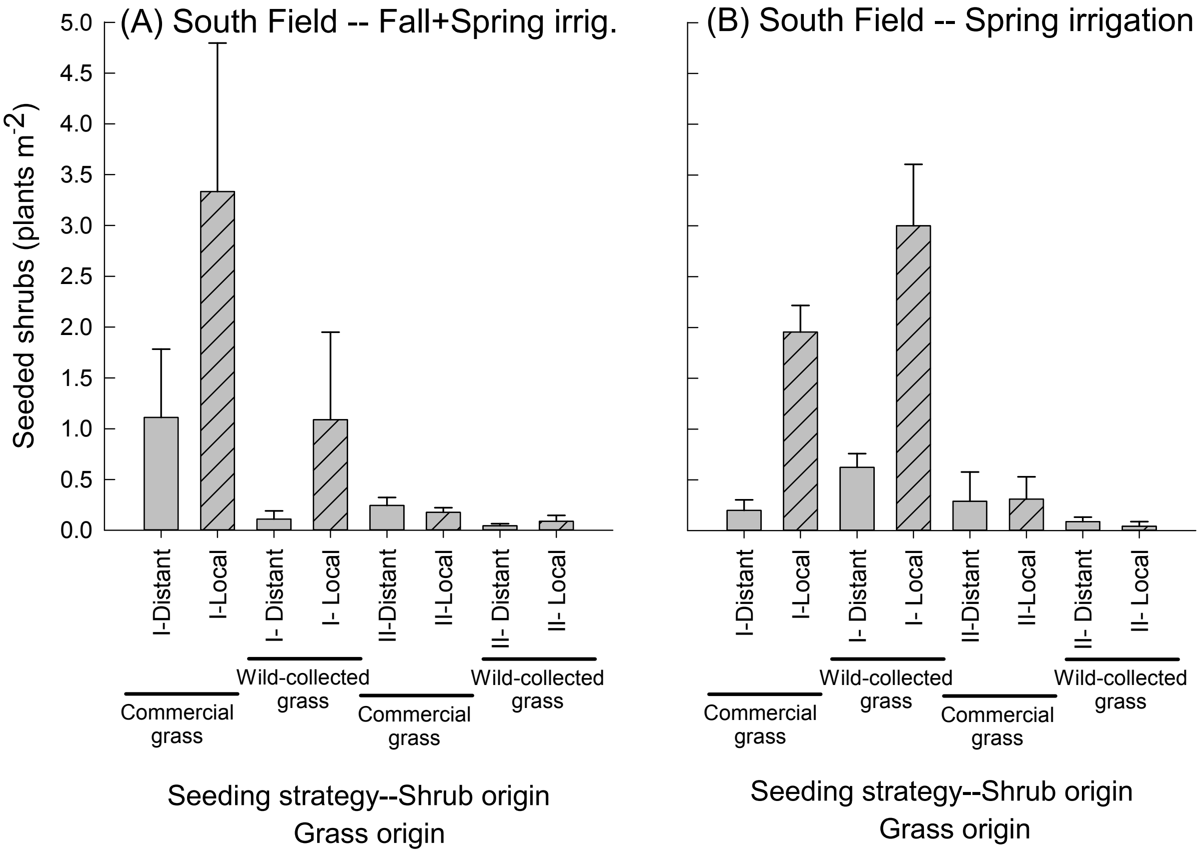

Supplement: S6 Appendix — (PDF) [file pone.0205760.s006.pdf]
